# Reusable Mesh Signature Scheme for Protecting Identity Privacy of IoT Devices

**DOI:** 10.3390/s20030758

**Published:** 2020-01-30

**Authors:** Ke Gu, WenBin Zhang, Se-Jung Lim, Pradip Kumar Sharma, Zafer Al-Makhadmeh, Amr Tolba

**Affiliations:** 1 School of Computer and Communication Engineering, Changsha University of Science and Technology, Changsha 410114, China; gk4572@163.com (K.G.);; 2 Liberal Arts & Convergence Studies, Honam University, Gwangju 62399, Korea; 3 Department of Multimedia Engineering, Dongguk University, Seoul 04620, Korea; pradip@dongguk.edu; 4 Computer Science Department, Community College, King Saud University, Riyadh 11437, Saudi Arabia; zalmakhadmee@ksu.edu.sa (Z.A.-M.); atolba@ksu.edu.sa (A.T.); 5 Mathematics and Computer Science Department, Faculty of Science, Menoufia University, Shebin-El-kom 32511, Egypt

**Keywords:** anonymity, mesh signature, IoT device, privacy, identity

## Abstract

The development of the Internet of Things (IoT) plays a very important role for processing data at the edge of a network. Therefore, it is very important to protect the privacy of IoT devices when these devices process and transfer data. A mesh signature (MS) is a useful cryptographic tool, which makes a signer sign any message anonymously. As a result, the signer can hide his specific identity information to the mesh signature, namely his identifying information (such as personal public key) may be hidden to a list of tuples that consist of public key and message. Therefore, we propose an improved mesh signature scheme for IoT devices in this paper. The IoT devices seen as the signers may sign their publishing data through our proposed mesh signature scheme, and their specific identities can be hidden to a list of possible signers. Additionally, mesh signature consists of some atomic signatures, where the atomic signatures can be reusable. Therefore, for a large amount of data published by the IoT devices, the atomic signatures on the same data can be reusable so as to decrease the number of signatures generated by the IoT devices in our proposed scheme. Compared with the original mesh signature scheme, the proposed scheme has less computational costs on generating final mesh signature and signature verification. Since atomic signatures are reusable, the proposed scheme has more advantages on generating final mesh signature by reconstructing atomic signatures. Furthermore, according to our experiment, when the proposed scheme generates a mesh signature on 10 MB message, the memory consumption is only about 200 KB. Therefore, it is feasible that the proposed scheme is used to protect the identity privacy of IoT devices.

## 1. Introduction

### 1.1. Background

**Figure 1:**
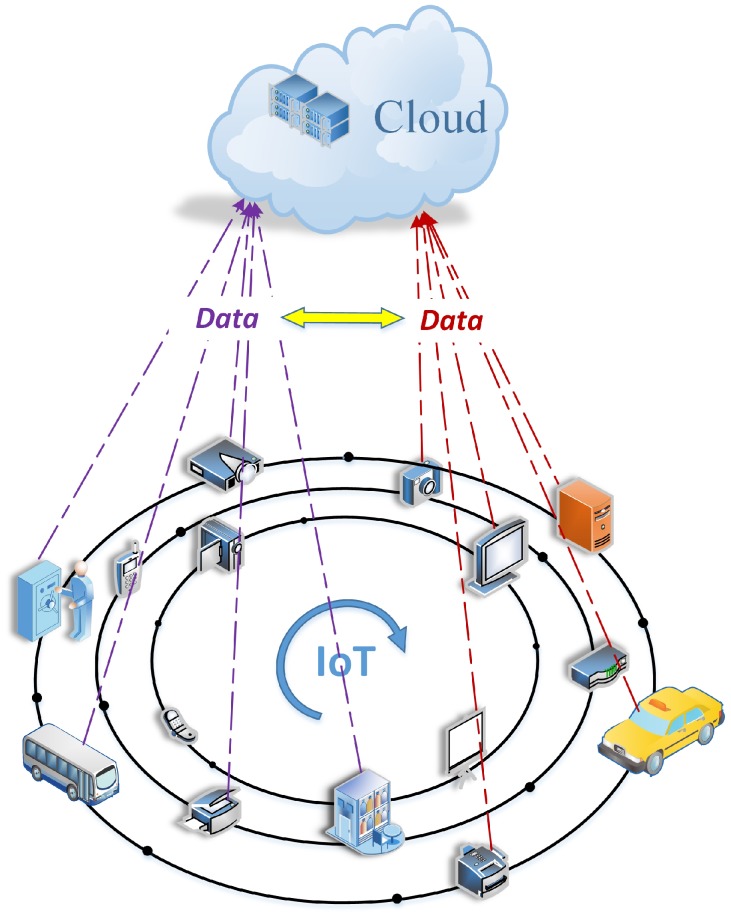
Data collection framework in IoT.

The Internet of Things (IoT) is an important environment for processing data at the edge of a network [1], where a huge amount of data is generated in IoT. Thus, we are always surrounded by IoT data in our homes, cars and offices. IoT devices are responsible for acquiring, storing and transferring data, as shown in Figure 1. By collecting, processing and analyzing the data through IoT devices, consumers and organizations can gain valuable insights, the data can further help them make better decisions for the future. However, since data usually comes from multiple IoT devices on different formats, after sensors acquire data from IoT devices, such as smart appliances, smart TVs, and wearable health devices, data must be preprocessed. In IoT, data may be transmitted, saved and retrieved at any time. For example, we build a system to collect location data of any things, such as a things track system. In the system, location data enables you to track your packages, pallets and devices in real time, rather than directing you to specific destinations. Therefore, as IoT devices keep “connected” and communicate with each other by introducing various new ways, IoT enables us to automatically complete certain tasks through some platforms, further making our life easier. Currently many IoT devices are located on the edge of a network and lack of protection measures to resist various attacks. Therefore, these devices are more vulnerable to some attacks, such as device theft, device manipulation, identity theft, data eavesdropping and so on. Once an IoT system is invaded, it may have a serious impact on the security of personal life or enterprise. For example, attackers may track a person by attacking his/her mobile phone; further, when a physical defense system based on IoT devices was successfully attacked in a building, it leads to that the attackers can more easily access some confidential areas in the building. Obviously the current vulnerabilities of IoT system can make attackers easier to implement these attacks. Therefore, when IoT devices process their data, their privacy is easily disclosed. It is very important to protect the privacy of IoT devices when these devices process and transfer data. Thus, the privacy of IoT devices needs to be focused. The privacy protection of IoT devices refers to the privacy protection measures to prevent the unnecessary disclosure of personal information. For the privacy protection technology of IoT devices, many scholars have done a lot of research. The current privacy protection technology mainly focuses on data publishing, data mining, wireless sensor network and other fields. In data publishing field, it is mainly divided into data distortion-based technology, data encryption-based technology and restricted publishing technology, among which the restricted publishing technology is mainly realized by data anonymity. For example, when IoT devices sign and publish their data, and the data anonymity technology may prevent disclosure of their identities. Additionally, IoT devices also need to publish a large amount of data, thus it is also very important for IoT devices to decrease the number of signatures generated by them in the same data. A mesh signature (MS) [2] allows a user to hide his specific identity information in a list of tuples that consist of public key and message when the user signs any message. Thus, mesh signature can only tell us that one of potential signers signed the message. Furthermore, a mesh signature consists of some atomic signatures, where the atomic signatures may be reused. Therefore, a mesh signature is a good choice for protecting the identities of IoT devices when these devices issue their data. For example, in some IoT devices that belong to one network group sign and publish their data through mesh signatures, no one can know the specific identities of the publishing IoT devices, and further the old mesh signatures are easily modified and reconstructed by partly generating some new atomic signatures so as to decrease the number of signatures.

A mesh signature is the extension of a ring signature [3]. Compared with ring signature, mesh signature can modularize the construction of signature, namely a user first must sign or collect enough atomic signatures which are seen as the basic elements of mesh signature, then the user may construct an access structure to mesh the atomic signatures and generate the final mesh signature. Boyen first proposed the notion of mesh signature in the Cryptology-EUROCRYPT, 2007, and a revised version [4] in the Journal of Cryptology, 2015. In the notion of mesh signatures, access structure is used to construct different combinations of atomic signatures; and mesh signature does not disclose that which atomic signature was used, thus atomic signatures can be reusable when a new mesh signature needs to be generated. Compared with a ring signature, a mesh signature has the modularity, which may provide much richer predicate expression of language. In [2,4], according to the context of mesh signature, the mesh signature may use a tree as the access structure to represent the relationship of atomic signatures. In the tree, its interior nodes denote the logic relationships, such as “And”, “Or”, and “Threshold gates”, and its leaf nodes denote the specific atomic signatures. Thus, the construction of mesh signature is similar to another anonymous signature, attribute-based signature (ABS) [5]. Compared with other kind of anonymous signatures (ring signature, attribute-based signature and group signature [6]), the mesh signature consists of some atomic signatures, where the atomic signatures can be reusable. Thus, the merit is very suitable for IoT devices. As IoT devices can generate a large amount of data every day, if each IoT device both needs to sign and then publish its data, then the signing cost is very heavy for itself, which needs to consume a lot of energy. However, for many IoT devices, some publishing data are the same. Thus, if each IoT device may reuse some “old” signatures by itself on the same data, then it will save the signing cost so as to decrease the number of signatures generated by IoT devices. Therefore, for a large amount of data published by the IoT devices, mesh signature is suitably used for publishing the same data.

**Figure 2:**
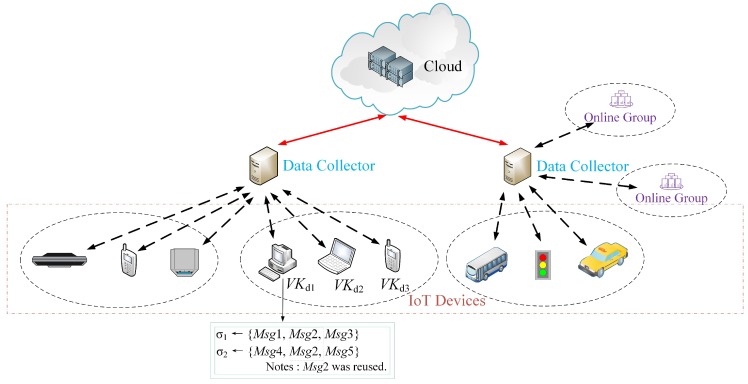
A example of mesh signature in IoT.

We have the following example to show that how the structure of mesh signature is used to protect the identities of IoT devices. For example, IoT device 1, IoT device 2 and IoT device 3 belong to a online group at the edge of the network, where the public verification key of IoT device 1 is $VK_{d1}$, the public verification key of IoT device 2 is $VK_{d2}$ and the public verification key of IoT device 3 is $VK_{d3}$. These devices both need to send their data to the IoT data collector, as shown in Figure 2. When the IoT device 1 issues a tuple of messages {Msg1, Msg2, Msg3} to the IoT data collector, it does not want to disclose that these messages are only published by itself. Therefore, this device may create such mesh signature, $\sigma_{1}=\underset{atomic\;signature-1}{\underbrace{\left[VK_{d1}:Msg1\right]}}\;And\;\underset{atomic\;signature-2}{\underbrace{\left[VK_{d2}:Msg2\right]}}\;And\;\underset{atomic\;signature-3}{\underbrace{\left[VK_{d3}:Msg3\right]}}$.

Then this device issues these messages by the names of three devices, thus its specific identity can be hidden into these names. Additionally, another feature of mesh signature is that it is modularized and its atomic signatures can be reusable, which is suitable for the same data published by the IoT devices. For example, IoT device 1 may flexibly create a new mesh signature on other messages {Msg4,Msg2,Msg5},

$$\sigma_{2}=\underset{atomic\;signature-4}{\underbrace{\left[VK_{d1}:Msg4\right]}}\;And\;\underset{atomic\;signature-2}{\underbrace{\left[VK_{d2}:Msg2\right]}}\;And\;\underset{atomic\;signature-5}{\underbrace{\left[VK_{d3}:Msg5\right]}},$$

where the atomicsignature−2 that binds to IoT device 2 is reused. As mesh signature has perfect anonymity, it does not disclose any fact that how the two signatures $\sigma_{1}$ and $\sigma_{2}$ are made up as long as the signatures $\sigma_{1}$ and $\sigma_{2}$ are valid.

However, although mesh signatures may be used in many security fields [3,7,8,9,10,11,12,13,14,15,16,17,18,19,20], few researchers focused on the improvement of mesh signatures because of their complexity. Currently the generation of mesh signatures consists of two main steps: (1) generating some atomic signatures; (2) generating a final mesh signature based on previous atomic signatures. Because atomic signatures can be reused, randomization technology is employed so that any adversary cannot know which atomic signatures were reused. Compared with other similar anonymous signature schemes, the generation of mesh signatures is relatively complicated in the existing schemes. In this paper, we focus on improving mesh signatures, where we construct a novel mesh signature scheme for IoT devices.

### 1.2. Our Contributions

In this paper, we present an improved mesh signature for protecting the identities of IoT devices. Also, we give a syntax of mesh signature in IoT. In this paper, our detailed contributions are as follows:

- We present a syntax for mesh signature in IoT. Compared with the works of [2,4], we further clearly describe the frame of mesh signature in IoT. Under the proposed syntax, we present a fully anonymous mesh signature scheme for IoT devices, where the IoT devices may be seen as the signers to sign their data and their specific identities can be hidden. Additionally, the atomic signatures on the same data can be reusable so as to decrease the number of signatures generated by IoT devices.
- In our proposed scheme, we have limitedly defined the access structure of language expression by monotone-span programs, thus the proposed mesh signature can resist the collusion attacks and its access structure still support generalized monotone predicates. Also, under the security frame proposed by [2,4], our proposed scheme is secure in the standard model, where the security of our scheme can be reduced to the CDH assumption. Also, the proposed scheme has the anonymity with enough security to protecting the identities of IoT devices.
- Compared with the original mesh signature scheme [2], the proposed scheme preserves the original modularity. Although generating atomic signatures in the proposed scheme needs more computational cost, the proposed scheme has less computational costs on generating final mesh signature and signature verification. Since atomic signatures are reusable, the proposed scheme has more advantages on generating final mesh signature by reconstructing atomic signatures. According to our experiment, it is feasible that the proposed scheme is used to protect the identity privacy of IoT devices.

### 1.3. Organization

The rest of this paper is organized as follows. In Section 2, we discuss the related works about the privacy protection of IoT devices. In Section 3, we review the complexity assumptions and the related technologies on which we build. In Section 4, we show a syntax for MS in IoT. In Section 5, we propose an improved mesh signature scheme for protecting the identities of IoT devices. In Section 6, we analyze the efficiency and security of the proposed scheme. Finally, we draw our conclusions in Section 7.

## 2. Related Work

Currently, many signature schemes have been used to protect the privacy (identities) of IoT devices. Li [21] proposed an attribute-based signature to receive WiFi beacons and use Doppler Effect and multipath signal to produce signatures. In their scheme, because these generated signatures do not need sensor attachments, the related identities are still anonymous. Karati [1] proposed a secure certificateless signature scheme to protect industrial-IoT Environments. The proposed signature scheme is proved to be secure under bilinear strong Diffie–Hellman (BSDH) assumptions, which can resist the Type-I and Type-II attacks. Furthermore, they analyzed the performance of their scheme, which is superior to other similar schemes. Sun [22] proposed a decentralized multi-authority attribute-based signature scheme for IoT devices. Compared with other similar signature schemes, their proposed scheme has more perfect privacy and can resist authority corruption. Furthermore, their scheme employs an extra cloud server to sign messages so as to decrease the signing cost. Xie [23] proposed a novel group signature based on lattice for anonymous authentication in IoT. In their scheme, a user may dynamically join a network group, and their proposed scheme easily revoke a group membership when the user quits the group. Also, their scheme can effectively resist the frameability attack, where other users cannot forge any user’s signature. Furthermore, their scheme is proved to be secure under lattice problem. Mughal [24] proposed a lightweight shortened signature scheme to secure the communication between devices in human centered IoT. In their scheme, the signing and verification procedures need less costs. Also, for different document protection requirements, their scheme provides the parameter selection function to make signature/verification. Their scheme is enough secure to resist traffic analysis attacks. Additionally, compared with other similar signature schemes, their scheme provides an experimental environment to test that whether their scheme can secure the communication procedure between cell phones (or smart devices). The obtained results show their scheme is effective. Cui [25] also proposed an attribute-based signature to protect industrial-IoT Environments under constrained resources. Their scheme employs a server to decrease the signing and verification cost, where a signing procedure can be immediately ceased when a signer is revoked. Li [26] proposed an effective ring signcryption scheme to protect the data transmission procedure from sensors to servers in IoT under public key infrastructure. They proved that their scheme is indistinguishable under adaptive chosen ciphertext attacks and unforgeable under adaptive chosen message attacks, whose security can be reduced to the computational Diffie–Hellman (CDH) assumption.

Additionally, many new anonymous signature schemes were also proposed, where the group signature [27,28,29,30,31], ring signature [32,33,34] and attribute-based signature [35,36,37] all belong to anonymous signatures. Libert et al. [28] proposed an effective group signature. Their proposed scheme has linear size public keys, linear size revocation list and constant signature size. Furthermore, the verification time is constant. We [31] proposed a traceable identity-based group signature, which employs verifier-local revocation to revoke users. Under the proposed security frame, the security of our scheme can be reduced to the CDH assumption. Yuen et al. [32] proposed a linkable ring signature, which is based on the logic operations, such as “and”, “or” and “threshold”. In their scheme, a sub-linear size $O(d\cdot \sqrt{n})$ signature can be generated, where *d* is a threshold and *n* is the number of potential signers in a ring. Liu et al. [33] also proposed a perfect anonymous linkable ring signature scheme, where the generated signature size is still linear with the number of possible signers in a ring. Au et al. [34] proposed a novel identity-based linkable ring signature scheme, which is revocable-iff-linked. Kaafarani et al. [35] proposed some traceable attribute-based signatures, which are decentralized. Their schemes provide anonymity under adaptive chosen-ciphertext attack. We [37] proposed an attribute-based signature, which supports monotone predicates. Compared with other similar schemes, our scheme is efficient by decreasing the signing and verification cost. Boyen first proposed the original mesh signature in [2], which may be seen as the extension of ring signature. Compared with other kind of anonymous signatures, the most advantage of mesh signature is that it can modularize the construction of signature and provide much richer predicate expression of language. In 2015, Boyen proposed a revised version in [4]. He considered that the construction of mesh signature is more flexible than that of ring signature, thus they proposed the notion of mesh signature, in which the access structure is used to construct different combinations of atomic signatures; and mesh signature does not disclose that which atomic signature was used, thus atomic signatures can be reusable when a new mesh signature needs to be generated. However, as the modularity of mesh signature is open to the construction of access structure of language expression, original mesh signature [2,4] has a security weakness that this scheme cannot satisfy the strict unforgeability because multiple illegal signers may collusively pool their obtained atomic signatures together and then generate final mesh signature which none of them could produce.

## 3. Preliminaries

### 3.1. Bilinear Maps

Let $G_{1}$ and $G_{2}$ be groups of prime order *q* and *g* be a generator of $G_{1}$. We say $G_{2}$ has an admissible bilinear map, $e:{G_{1}\times G_{1}\to G}_{2}$ if the following two conditions hold. The map is bilinear; for all *a*, *b*, we have $e\left(g^{a},g^{b}\right)=e{\left(g,g\right)}^{a\cdot b}$. The map is non-degenerate; we must have that $e\left(g,g\right)\neq 1$.

### 3.2. Computational Diffie–Hellman Assumption

**Definition 1** **(Computational Diffie–Hellman (CDH) Problem).** *Let $G_{1}$ be a group of prime order q and g be a generator of $G_{1}$; for all $\left(g,g^{a},g^{b}\right)\in G_{1}$, with $a,b\in Z_{q}$, the CDH problem is to compute $g^{a\cdot b}$.*

**Definition** **2.** *The (ℏ,ε)-CDH assumption holds if no ℏ-time algorithm can solve the CDH problem with probability at least ε.*

### 3.3. Monotone-Span Programs

Let $\Upsilon :{\left\{0,1\right\}}^{n}\to \left\{0,1\right\}$ be a monotone boolean function. A monotone span program [5] for Υ over a field F is an l×t matrix Λ with entries in F, along with a labeling function ϖ:[l]→[n] that associates each row of Λ with an input variable of Υ, that, for every ($x_{1}$, $x_{2}$……$x_{n}$)$\in {\left\{0,1\right\}}^{n}$, satisfies the following:

$$\Upsilon \left(x_{1},\ldots \ldots x_{n}\right)=1\Leftrightarrow \exists \vec{\eta }\in F^{1\times l}:\vec{\eta }\cdot \Lambda =\left\{1,0,0\ldots \ldots 0\right\}\;and\;\left(\forall i:x_{\varpi (i)}=0\Rightarrow \eta_{i}=0\right);$$

in other words, Υ($x_{1}$, $x_{2}$……$x_{n}$)=1 if and only if the rows of Λ indexed by $\left\{i|x_{\varpi (i)}=1\right\}$ span the vector [1,0,0……0], where we call *l* the length and *t* the width of the span program, and l+t the size of the span program.

## 4. A Syntax for MS in IoT

**Figure 3:**
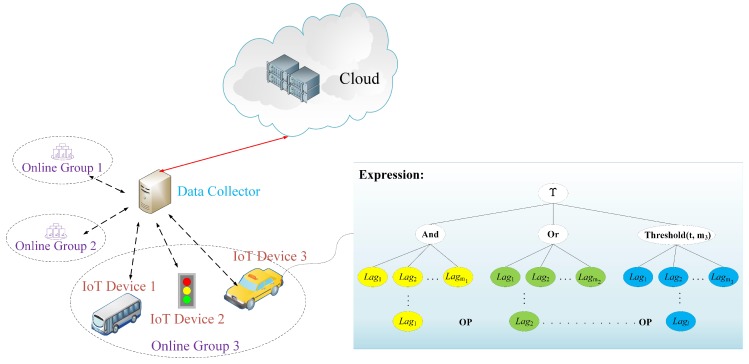
Syntax for mesh signature in IoT.

In this section, we present a syntax for mesh signature in IoT, where each IoT device is seen as a signer, they need to issue their data to the IoT data collector. Intuitively, a mesh signature is the combination of some atomic signatures, which satisfies the condition that the monotone boolean expression Υ over access structure (or expression structure) is true. Therefore, in our proposed syntax we set that the monotone boolean expression Υ is associated with a list of tuples that consist of public key and message and its value is true if one IoT device possesses some corresponding atomic signatures on the verified messages under the public verification keys, as shown in Figure 3.

In Figure 3, when one IoT device belonging to a network group needs to issue its data set to the IoT data collector, the whole language expression Expression is represented by the form $Expression::=\{Lag_{1}\;\mathrm{OP}\;Lag_{2}\ldots \ldots \mathrm{OP}\;Lag_{l}\}$, where $Lag_{i}$ is sub-expression belongs to the whole expression, OP denotes the operation on the sub-expressions, *l* is the number of involved IoT devices belonging to the same network group (or the number of atomic clauses in a mesh structure). The more detailed and generalized form is as follows:

$$Expression::=\{Lag_{1}\;\mathrm{OP}\;Lag_{2}\ldots \ldots \mathrm{OP}\;Lag_{l}\}$$

$$=\;\mathrm{And}\{Lag_{1}\;,\;Lag_{2}\ldots \ldots \;Lag_{m_{1}}\}$$

$$\left|\;\mathrm{Or}\{Lag_{1}\;,\;Lag_{2}\ldots \ldots \;Lag_{m_{2}}\right\}$$

$$|\;{\mathrm{Threshold}}_{t,m_{3}}\left\{Lag_{1}\;,\;Lag_{2}\ldots \ldots \;Lag_{m_{3}}\right\},$$

where we set $l=m_{1}+m_{2}+m_{3}$. Then we consider the monotone boolean expression Υ over access structure is true only if $\Upsilon (Lag_{1},Lag_{2}\ldots \ldots Lag_{l})=1$. Thus, for the previous-mentioned example, $\sigma_{1}=\underset{atomic\;signature-1}{\underbrace{\left[VK_{d1}:Msg1\right]}}\;And\;\underset{atomic\;signature-2}{\underbrace{\left[VK_{d2}:Msg2\right]}}\;And\;\underset{atomic\;signature-3}{\underbrace{\left[VK_{d3}:Msg3\right]}}$, the form of the atomic signature $\left[VK_{i}:Msg_{i}\right]$ is set to $Lag_{i}$, which means this “$Msg_{i}$” is signed under $VK_{i}$.

**Definition** **3.** *Improved Mesh signature in IoT: Let **MS** = (**System-Setup**, **Generate-Key**, **Mesh-Sign**, **Mesh-Verify**) be a mesh signature scheme in IoT. In **MS**, all detailed algorithms are as follows:*

**(1)**  ***System-Setup**: The authority system runs the randomized algorithm, and inputs a security parameter $1^{k}$. In addition, the algorithm outputs all related public system parameters MRK and a master system private key msk on the parameter $1^{k}$.*
**(2)**  ***Generate-Key**: The authority system runs the randomized algorithm, and inputs (MRK, msk), and then outputs the IoT device’s private/public key pair $\left(sk_{i},pk_{i}\right)$ to the device i, where i∈{1,2……,n} (we set that n is the number of the IoT devices).*
**(3)**  **Mesh-Sign**: The randomized algorithm generates a mesh signature. The IoT device i issues its message set (data) $M\in {\left\{0,1\right\}}^{*}$ and then signs the message set, thus the device i runs the algorithm: (a) the algorithm inputs (MRK, $sk_{i}$, PK_List, M), and then outputs a monotone boolean expression *Υ* and the atomic signatures $\sigma_{i}$; (b) the algorithm inputs (MRK, $sk_{i}$, $\sigma_{i}$, *Υ*), and then outputs a mesh signature *Φ*, where PK_List is a list of all the public keys of the devices involved with this signing; (c) the algorithm run by the device i sends the message set M, the boolean expression *Υ* and the mesh signature *Φ* to the IoT data collector.
**(4)**  ***Mesh-Verify**: The IoT data collector verifies the standard mesh signature *Φ* on Υ and M. The IoT data collector runs the deterministic algorithm, and inputs (MRK, PK_List, M, *Υ*, *Φ*), and then outputs the result, accept or reject.*

## 5. Improved Mesh Signature Scheme for IoT Devices

**Figure 4:**
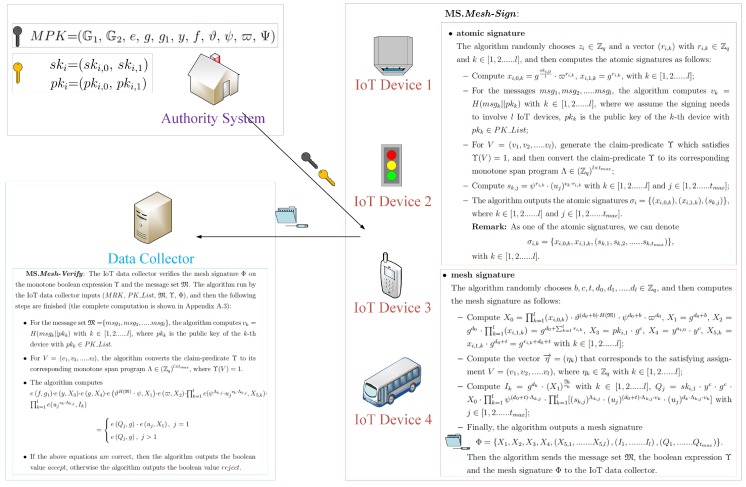
Improved mesh signatures for IoT devices.

In the section, we propose an improved mesh signature scheme for protecting the identities of IoT devices. Currently the generation of mesh signatures consists of two main steps: (1) generating some atomic signatures; (2) generating a final mesh signature based on previous atomic signatures. Because atomic signatures can be reused, in our construction the randomization technology is also employed so that any adversary cannot know which atomic signatures were reused. Compared with the original mesh signature [2,4], we have limitedly defined the access structure of language expression by monotone-span programs, thus improved mesh signature can still support generalized monotone predicates over access structure. Let **MS** = (***System-Setup***, ***Generate-Key***, ***Mesh-Sign***, ***Mesh-Verify***) be a mesh signature scheme in IoT. In **MS**, all detailed algorithms are described as follows (shown in Figure 4):

**(1)**  **MS.*****System-Setup***: The system runs this setup algorithm, and inputs the parameter $1^{k}$ (used as the security level). Also, we set that $G_{1}$ and $G_{2}$ are the groups of prime order *q*, *g* is a generator of $G_{1}$, and that $e:{G_{1}\times G_{1}\to G}_{2}$ denotes the bilinear map. In addition, we set that $H:{\left\{0,1\right\}}^{*}\to Z_{1^{k}\cdot q}$ denotes one hash function and it can be used to output integers in $Z_{1^{k}\cdot q}$. Additionally, we assume that the monotone span programs related to claim-predicates have their width at most $t_{max}$ in our construction.
  Then the following parameters are outputted in the system. The algorithm randomly chooses $a\in Z_{q}$ and sets $g_{1}=g^{a}$. Five group elements *y*, *f*, ϑ, ψ and $\varpi \in G_{1}$ are randomly picked. Also, the algorithm generates a $t_{max}$-length vector $\Psi =(u_{i})$, whose element $u_{i}$ is randomly picked from $G_{1}$. Finally the algorithm outputs the public parameters MPK = ($G_{1}$, $G_{2}$, *e*, *g*, $g_{1}$, *y*, *f*, ϑ, ψ, ϖ, Ψ), where msk=a is a master private key in the system.
**(2)**  **MS.*****Generate-Key***: The system runs the algorithm and then generates IoT device’s private/public key pair. For the device *i*, the algorithm inputs (MRK, msk), and then it randomly picks $a_{i,0},a_{i,1}\in Z_{q}$, sets $sk_{i,0}=a_{i,0}$ and computes $sk_{i,1}=f^{msk}\cdot y^{a_{i,1}}=f^{a}\cdot y^{a_{i,1}}$, $pk_{i,0}=g^{a_{i,0}}$ and $pk_{i,1}=g^{a_{i,1}}$, where we set $sk_{i}$ = ($sk_{i,0}$, $sk_{i,1}$) as the private key of the device *i* and $pk_{i}$ = ($pk_{i,0}$, $pk_{i,1}$) as the public key of the device *i*.
**(3)**  **MS.*****Mesh-Sign***: The IoT device *i* signs a message set $M\in {\left\{0,1\right\}}^{*}$, where the message set M=$\left\{msg_{1},msg_{2},\ldots \ldots ,msg_{l}\right\}$. The device *i* runs the algorithm, and then inputs (MRK, $sk_{i}$, PK_List, M) where PK_List is a list of the public keys of the IoT devices involved with this signing, and then the following steps are finished:
  - **atomic signature**
    The algorithm randomly chooses $z_{i}\in Z_{q}$ and a vector ($r_{i,k}$) with $r_{i,k}\in Z_{q}$ and k∈[1,2……l], and then computes the atomic signatures as follows:
    - Compute $x_{i,0,k}=g^{\frac{sk_{i,0}}{l}}\cdot \varpi^{r_{i,k}}$, $x_{i,1,k}=g^{r_{i,k}}$, with k∈[1,2,……,l];
    - For the messages $msg_{1},msg_{2},\ldots \ldots ,msg_{l}$, the algorithm computes $v_{k}=H(msg_{k}||pk_{k})$ with k∈[1,2……,l], where we assume the signing needs to involve *l* IoT devices, $pk_{k}$ is the public key of the *k*-th device with $pk_{k}\in PK\_List$;
    - For $V=(v_{1},v_{2},\ldots \ldots ,v_{l})$, generate the claim-predicate Υ which satisfies Υ(V)=1, and then transform the claim-predicate Υ to its corresponding monotone span program $\Lambda \in {\left(Z_{q}\right)}^{l\times t_{max}}$;
    - Compute $s_{k,j}=\psi^{r_{i,k}}\cdot {\left(u_{j}\right)}^{v_{k}\cdot r_{i,k}}$ with k∈[1,2……,l] and $j\in [1,2\ldots \ldots ,t_{max}]$;
    - The algorithm outputs the atomic signatures $\sigma_{i}=\left\{\left(x_{i,0,k}\right),\left(x_{i,1,k}\right),\left(s_{k,j}\right)\right\}$, where k∈[1,2……,l] and $j\in [1,2\ldots \ldots ,t_{max}]$.
      **Remark.**  As one of the atomic signatures, we can denote
      $$\sigma_{i,k}=\left\{x_{i,0,k},x_{i,1,k},\left(s_{k,1},s_{k,2},\ldots \ldots ,s_{k,t_{max}}\right)\right\},$$
      with k∈[1,2……,l].
  - **mesh signature**
    The algorithm randomly chooses $b,c,t,d_{0},d_{1},\ldots \ldots ,d_{l}\in Z_{q}$, and then computes the mesh signature as follows:
    - Compute $X_{0}=\prod_{k=1}^{l}\left(x_{i,0,k}\right)\cdot \vartheta^{\left(d_{0}+b\right)\cdot H\left(M\right)}\cdot \psi^{d_{0}+b}\cdot \varpi^{d_{0}}$, $X_{1}=g^{d_{0}+b}$, $X_{2}=g^{d_{0}}\cdot \prod_{k=1}^{l}\left(x_{i,1,k}\right)=g^{d_{0}+\sum_{k=1}^{l}r_{i,k}}$, $X_{3}=pk_{i,1}\cdot g^{c}$, $X_{4}=y^{a_{i,0}}\cdot y^{c}$, $X_{5,k}=x_{i,1,k}\cdot g^{d_{0}+t}=g^{r_{i,k}+d_{0}+t}$ with k∈[1,2……,l];
    - Compute the vector $\vec{\eta }=\left(\eta_{k}\right)$ related to the satisfying assignment $V=(v_{1},v_{2},\ldots \ldots ,v_{l})$, where $\eta_{k}\in Z_{q}$ with k∈[1,2……,l];
    - Compute $I_{k}=g^{d_{k}}\cdot {\left(X_{1}\right)}^{\frac{\eta_{k}}{v_{k}}}$ with k∈[1,2……,l], $Q_{j}=sk_{i,1}\cdot y^{c}\cdot g^{c}\cdot X_{0}\cdot \prod_{k=1}^{l}\psi^{\left(d_{0}+t\right)\cdot \Lambda_{k,j}}\cdot \prod_{k=1}^{l}\left[{\left(s_{k,j}\right)}^{\Lambda_{k,j}}\cdot {\left(u_{j}\right)}^{\left(d_{0}+t\right)\cdot \Lambda_{k,j}\cdot v_{k}}\cdot {\left(u_{j}\right)}^{d_{k}\cdot \Lambda_{k,j}\cdot v_{k}}\right]$ with $j\in [1,2\ldots \ldots ,t_{max}]$;
    - The algorithm finally generates and outputs a mesh signature
      $\Phi =\{X_{1},X_{2},X_{3},X_{4},\left(X_{5,1},\ldots \ldots ,X_{5,l}\right),\left(I_{1},\ldots \ldots ,I_{l}\right),\left(Q_{1},\ldots \ldots ,Q_{t_{max}}\right)\}.$ Then the algorithm sends the message set M, the boolean expression Υ and the mesh signature Φ to the IoT data collector.
**(4)**  **MS.*****Mesh-Verify***: The IoT data collector verifies the mesh signature Φ on the monotone boolean expression Υ and the message set M. The algorithm run by the IoT data collector inputs (MRK, PK_List, M, Υ, Φ), and then the following steps are finished (the complete computation is shown in Appendix A.3):
  - For the message set M=$\left\{msg_{1},msg_{2},\ldots \ldots msg_{l}\right\}$, the algorithm computes $v_{k}=H(msg_{k}||pk_{k})$ with k∈[1,2……,l], where $pk_{k}$ is the public key of the *k*-th device with $pk_{k}\in PK\_List$.
  - For $V=(v_{1},v_{2},\ldots \ldots ,v_{l})$, the algorithm transforms the claim-predicate Υ to the monotone span program $\Lambda \in {\left(Z_{q}\right)}^{l\times t_{max}}$, where Υ(V)=1.
  - The algorithm computes $e\left(f,g_{1}\right)\cdot e\left(y,X_{3}\right)\cdot e\left(g,X_{4}\right)\cdot e\left(\vartheta^{H(M)}\cdot \psi ,X_{1}\right)\cdot e\left(\varpi ,X_{2}\right)\cdot \prod_{k=1}^{l}e\left(\psi^{\Lambda_{k,j}}\cdot {u_{j}}^{v_{k}\cdot \Lambda_{k,j}},X_{5,k}\right)\cdot \prod_{k=1}^{l}e\left({u_{j}}^{v_{k}\cdot \Lambda_{k,j}},I_{k}\right)$
    $$=\left\{\begin{array}{c} e\left(Q_{j},g\right)\cdot e\left(u_{j},X_{1}\right),\;j=1\\ e\left(Q_{j},g\right),\;j>1 \end{array}\right.$$
    If the equation is correct, the algorithm outputs accept, otherwise it outputs reject.

## 6. Analysis of Our Scheme

### 6.1. Security Analysis

In our proposed mesh signature scheme, we need to consider the two notions “one-more unforgeability” and “full anonymity”. First, any IoT device cannot forge a new mesh signature on any corrupted or fresh information. Second, the anonymity of IoT device will be preserved even if some atomic signatures are reused to generate a new mesh signature, namely mesh signature and its atomic signatures must be anonymous, where we need to use the technology of randomization to randomize the generated signatures. Under the security frame proposed by [2,4], our scheme is proven to be unforgeable and anonymous.

**Theorem** **1.** *Our proposed scheme is (ℏ, ε, $q_{k}$, $q_{a}$, $q_{m}$)-unforgeable, where we assume that the ($\hbar^{\prime }$, $\varepsilon^{\prime }$)-CDH assumption can hold in $G_{1}$, and:*

$$\begin{array}{c} \varepsilon^{\prime }=\left(1-\frac{q_{k}}{q}\right)\cdot \left[1-q_{a}+q_{a}\cdot {\left(1-\frac{1}{q}\right)}^{l}\right]\cdot \left(1-\frac{q_{m}}{q}\right)\cdot \varepsilon ,\\ \hbar^{\prime }=\hbar +O(q_{k}\cdot \left[3\cdot C_{exp}+C_{mul}\right]+q_{a}\cdot \left[\left(2\cdot l\cdot t_{max}+3\right)\cdot C_{exp}+\left(l\cdot t_{max}+1\right)\cdot C_{mul}\right]+q_{m}\cdot [(4\cdot l\cdot t_{max}+\\ 3\cdot l+13)\cdot C_{exp}+\left(4\cdot l\cdot t_{max}+4\cdot l+8\right)\cdot C_{mul}]), \end{array}$$

*$q_{k}$ denotes the queries number of “Generate-Key” oracle, $q_{a}$ denotes the queries number of “Atomic Signature” oracle, $q_{m}$ denotes the queries number of “Mesh Signature” oracle, $C_{mul}$ denotes the time of a multiplication in $G_{1}$, $C_{exp}$ denotes the time of an exponentiation in $G_{1}$.*(This proof is provided to Appendix A.1)

**Theorem** **2.** *Our proposed scheme is (ℏ, ε, $q_{k}$, $q_{a}$, $q_{m}$)-anonymous, where we assume that the ($\hbar^{\prime }$, $\varepsilon^{\prime }$)-CDH assumption can hold in $G_{1}$, and:*

$$\begin{array}{c} \varepsilon^{\prime }=\left(1-\frac{q_{k_{1}}}{q}\right)\cdot \left(1-\frac{q_{k_{2}}}{q}\right)\cdot \left[1-q_{a_{1}}+q_{a_{1}}\cdot {\left(1-\frac{1}{q}\right)}^{l}\right]\cdot \left[1-q_{a_{2}}+q_{a_{2}}\cdot {\left(1-\frac{1}{q}\right)}^{l}\right]\cdot \left(1-\frac{q_{m_{1}}}{q}\right)\cdot \left(1-\frac{q_{m_{2}}}{q}\right)\cdot \left(\varepsilon -\frac{1}{2}\right),\\ \hbar^{\prime }=\hbar +O(\left(q_{k_{1}}+q_{k_{2}}\right)\cdot \left[3\cdot C_{exp}+C_{mul}\right]+\left(q_{a_{1}}+q_{a_{2}}\right)\cdot \left[\left(2\cdot l\cdot t_{max}+3\right)\cdot C_{exp}+\left(l\cdot t_{max}+1\right)\cdot C_{mul}\right]+\\ \left(q_{m_{1}}+q_{m_{2}}\right)\cdot \left[\left(4\cdot l\cdot t_{max}+3\cdot l+13\right)\cdot C_{exp}+\left(4\cdot l\cdot t_{max}+4\cdot l+8\right)\cdot C_{mul}\right]), \end{array}$$

*$q_{k_{1}}$ and $q_{k_{2}}$ denote the queries numbers of “Generate-Key” oracle in the query phases 1 and 2 respectively, $q_{a_{1}}$ and $q_{a_{2}}$ denote the queries numbers of “Atomic Signature” oracle in the query phases 1 and 2 respectively, $q_{m_{1}}$ and $q_{m_{2}}$ denote the queries numbers of “Mesh Signature” oracle in the query phases 1 and 2 respectively, $C_{mul}$ denotes the time of a multiplication in $G_{1}$, $C_{exp}$ denotes the time of an exponentiation in $G_{1}$.*(This proof is provided to Appendix A.2)

### 6.2. Efficiency Analysis

In the proposed scheme, the length of the atomic signatures is $\left(2\cdot l+l\cdot t_{max}\right)\cdot \left|G_{1}\right|$, the length of the mesh signature is $\left(4+2\cdot l+t_{max}\right)\cdot \left|G_{1}\right|$, where $|G_{1}|$ is the size of element in $G_{1}$. Because $x_{i,0,k}$, $x_{i,1,k}$, $\psi^{r_{i,k}}$ in $s_{k,j}$ may be pre-computed (To make our analysis simple, we set the time of integer and hash computations is ignored.), signing a message set for the atomic signatures only computes at most $l\cdot t_{max}$ exponentiations in $G_{1}$ and $l\cdot t_{max}$ multiplications in $G_{1}$. Also, because $X_{1}$, $X_{2}$, $X_{3}$, $X_{4}$, $X_{5,k}$, $g^{d_{k}}$ in $I_{k}$, $\prod_{k=1}^{l}\left(x_{i,0,k}\right)\cdot \psi^{d_{0}+b}\cdot \varpi^{d_{0}}$ in $X_{0}$, $sk_{i,1}\cdot y^{c}\cdot g^{c}$ in $Q_{j}$ may be pre-computed, signing a message set for the mesh signature only computes at most $4\cdot l\cdot t_{max}+l+1$ exponentiations in $G_{1}$ and $4\cdot l\cdot t_{max}+l+1$ multiplications in $G_{1}$. In the verify algorithm, because the value $e(f,g_{1})$ can be pre-computed and cached, the verification needs $\left(2\cdot l+1\right)\cdot t_{max}+5$ pairing computations, $2\cdot l\cdot t_{max}$ exponentiations in $G_{1}$, $2\cdot l\cdot t_{max}+5$ multiplications in $G_{1}$. Furthermore, we compare our proposed scheme with the original mesh signature scheme [2] in detail. Table 1 shows the performance comparison according to our theoretical analysis (In this comparison, we assume that the order of assigned structure tree in [2] is set to $t_{max}$.), where $C_{mul}$ denotes the time of a multiplication in $G_{1}$, $C_{exp}$ denotes the time of an exponentiation in $G_{1}$ and $C_{pair}$ denotes the time of a pairing computation. According to Table 1, we can know although generating atomic signatures in our scheme needs more computational cost, our scheme has less computational costs on generating final mesh signature and signature verification. Since atomic signatures are reusable, our scheme has more advantages on generating final mesh signature by reconstructing atomic signatures.

Additionally, we make some experiments to test and evaluate the actual performance of our scheme. In the tests, we employ the paring based cryptography (PBC) library to simulate our scheme, where the experimental computer is under Intel Core i5 2.7 GHz and RAM 8GB. In our experiments, we use the Type A parings in PBC library to construct the parings, where the lengths of the parameters *p* and *q* are respectively set as 160 bits and 512 bits. Furthermore, the parameter *l* is set to {1, 10, 20, 30, 40, 50}, and then we test our scheme and the original scheme [2] 10 times on average under the different settings of *l*. Table 2 shows the actual performance comparison of our scheme and the original scheme. Similar to our theoretical analysis, our scheme has less computational costs on generating final mesh signature and signature verification, compared with the original scheme.

**Figure 5:**
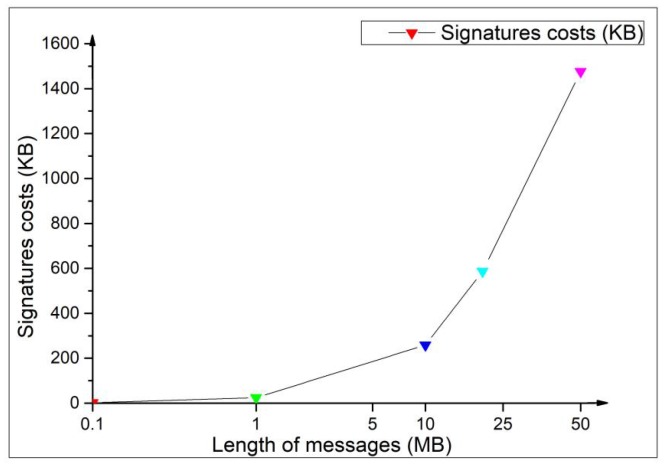
Memory consumption under different sizes of messages.

Since our scheme is used to protect the identity privacy of IoT devices, we further test our memory consumption through signing different sizes of messages. Figure 5 shows the change of memory consumption by signing different sizes of messages, where the sizes of messages are set to 100 KB, 1 MB, 10 MB, 20 MB, 50 MB respectively. In Figure 5, when our scheme generates a mesh signature on 10 MB message, the memory consumption is only about 200 KB. Therefore, it is feasible that our scheme is used to protect the identity privacy of IoT devices.

## 7. Conclusions

IoT devices are responsible for acquiring, storing, and transferring data. Currently, many IoT devices are located on the edge of a network and lack of protection measures to resist various attacks [38,39,40,41,42,43]. Therefore, these devices are more vulnerable to some attacks, such as device theft, device manipulation, identity theft, data eavesdropping and so on. Thus, the privacy of IoT devices needs to be focused. It is very important to protect the identities of IoT devices when these devices process and transfer data [44,45,46,47,48,49,50,51,52]. Then we present a syntax about mesh signature in IoT. Under the proposed syntax, we present a fully anonymous mesh signature scheme for IoT devices, where the IoT devices may be seen as the signers to sign their data and their specific identities can be hidden. In our proposed scheme, the generation of mesh signatures consists of two main steps: (1) generating some atomic signatures; (2) generating a final mesh signature based on previous atomic signatures. Additionally, as IoT devices can generate a large amount of data every day, if each IoT device both needs to sign and then publish its data, then the signing cost is very heavy for itself. Thus, if each IoT device reuses some “old” signatures by itself on the same data, it will save the signing cost so as to decrease the number of signatures generated by IoT devices. In our proposed scheme, the atomic signatures on the same data can be reusable so as to decrease the number of signatures. Although the atomic signatures can be reused, the randomization technology is employed so that any adversary cannot know which atomic signatures were reused. Thus, the merit is very suitable for IoT devices. Furthermore, in our proposed scheme we have limitedly defined the access structure of language expression by monotone-span programs, thus the proposed mesh signature can resist the collusion attacks and its access structure still support generalized monotone predicates. Compared with the original mesh signature scheme, our proposed scheme has its advantage, which has linear size length of signature.

## Figures and Tables

**Table 1:** Complexity of Two Schemes.

	Atomic Signatures	Mesh Signature	Verification
Original scheme [2]	$C_{exp}$	$\left(6\cdot \left(l+1\right)\cdot t_{max}\right)\cdot C_{exp}+$	$\left(\left(l+1\right)\cdot t_{max}+1\right)\cdot C_{pair}+$
		$\left(4\cdot l\cdot t_{max}+t_{max}\right)\cdot C_{mul}$	$3\cdot \left(l+1\right)\cdot t_{max}\cdot C_{exp}+$
			$3\cdot l\cdot t_{max}\cdot C_{mul}$
Our scheme	$l\cdot t_{max}\cdot \left(C_{exp}+C_{mul}\right)$	$(4\cdot l\cdot t_{max}+l+1)\cdot$	$\left(\left(2\cdot l+1\right)\cdot t_{max}+5\right)\cdot C_{pair}+$
		$\left(C_{exp}+C_{mul}\right)$	$2\cdot l\cdot t_{max}\cdot C_{exp}+$
			$\left(2\cdot l\cdot t_{max}+5\right)\cdot C_{mul}$

**Table 2:** Actual Performance of Two Schemes.

		Computational Costs (ms)					
1		1	10	20	30	40	50
Original scheme [2]	Atomic Signatures	1.958	1.746	1.590	1.605	1.566	1.629
	Mesh Signature	91.495	583.225	1038.55	1617.78	2003.15	2270.82
	Verification	61.457	339.048	593.315	1001.73	1263.15	1473.34
Our scheme	Atomic Signatures	7.890	78.850	164.900	236.100	313.600	387.250
	Mesh Signature	37.752	353.003	881.153	981.836	1551.64	1675.29
	Verification	37.910	441.830	591.710	1000.43	1150.84	1128.29

## Appendix A.

### Appendix A.1. Unforgeability

**Proof** **(Proof of Theorem 1).** We set that **MS** is our proposed mesh signature scheme. Also we set that A is an adversary with the tuple (*ℏ*, ε, $q_{k}$, $q_{a}$, $q_{m}$) that can make attack to **MS**. To make interaction with the adversary A, an algorithm B is constructed. For (*g*, $g^{a}$, $g^{b}$)$\in G_{1}$, B may make interaction with A to compute $g^{a\cdot b}$. Then the algorithm B can be assumed to solve the CDH problem with probability at least $\varepsilon^{\prime }$ and in time at most $\hbar^{\prime }$, which is contrary to the ($\hbar^{\prime }$, $\varepsilon^{\prime }$)-CDH assumption. Therefore, we may build a simulation procedure as follows: **Setup**: The parameter $1^{k}$ is inputted. Also, we set that $G_{1}$ and $G_{2}$ are the groups of prime order *q*, *g* is a generator of $G_{1}$, and that $e:{G_{1}\times G_{1}\to G}_{2}$ denotes the bilinear map. In addition, we set that $H:{\left\{0,1\right\}}^{*}\to Z_{1^{k}\cdot q}$ denotes one hash function and it can be used to output integers in $Z_{1^{k}\cdot q}$. Additionally, we assume that the monotone span programs related to claim-predicates have their width at most $t_{max}$ in our construction. Then the following parameters are outputted. The algorithm sets $g_{1}=g^{a}$ and $f=g^{b}$ with $a,b\in Z_{q}$ (B does not know *a* and *b*), chooses $\omega ,\beta ,\iota ,\varphi ,\varrho \in Z_{q}$, and then sets $y=f^{\omega }=g^{\beta }$, $\vartheta =g^{\iota }$, $\psi =g^{\varphi }$ and $\varpi =g^{\varrho }$. In addition, the algorithm chooses $\ell_{j}\in Z_{q}$ for all *j*s with $j\in [1,2\ldots \ldots t_{max}]$, and then sets $u_{j}=g^{\ell_{j}}$ for all *j*s with $j\in [1,2\ldots \ldots t_{max}]$. Then this system outputs all the parameters MRK = ($G_{1}$, $G_{2}$, *e*, *g*, $g_{1}$, *y*, *f*, ϑ, ψ, ϖ, $\Psi =(u_{j})$), where msk=a is seen as the master key of the system. **Queries**: A makes the following key and signature queries, then B gives its answers as follows:

- ***Generate-Key***(): Given the public parameters MRK, for the device *i*, the algorithm randomly chooses $a_{i,0},a_{i,1}\in Z_{q}$, sets $sk_{i,0}=a_{i,0}$ and computes $sk_{i,1}=y^{a_{i,1}}$, $pk_{i,0}=g^{a_{i,0}}$ and $pk_{i,1}=g^{a_{i,1}}\cdot g_{1}^{-\frac{1}{\omega }}$, where $sk_{i}$ = ($sk_{i,0}$, $sk_{i,1}$) is the private key of the device *i* and $pk_{i}$ = ($pk_{i,0}$, $pk_{i,1}$) is the public key of the device *i*, and then the private/public key pair is passed to the adversary A.
  **Remark****.** 
  To the correctness of $sk_{i}$ and $pk_{i}$, they may be changed as follows:
  $$\begin{array}{l} sk_{i,1}=y^{a_{i,1}}=f^{a}\cdot f^{-a}\cdot y^{a_{i,1}}=f^{a}\cdot f^{\omega \cdot \frac{-a}{\omega }}\cdot y^{a_{i,1}}=f^{a}\cdot y^{-\frac{a}{\omega }}\cdot y^{a_{i,1}}=f^{a}\cdot y^{a_{i,1}-\frac{a}{\omega }},\\ pk_{i,1}=g^{a_{i,1}}\cdot g_{1}^{-\frac{1}{\omega }}=g^{a_{i,1}}\cdot g^{-\frac{a}{\omega }}=g^{a_{i,1}-\frac{a}{\omega }} \end{array}$$
  Setting $a_{i,1}^{\prime }=a_{i,1}-\frac{a}{\omega }$, then $sk_{i,1}=f^{a}\cdot y^{a_{i,1}^{\prime }}$ and $pk_{i,1}=g^{a_{i,1}^{\prime }}$. Therefore, $sk_{i}$ and $pk_{i}$ is a valid private/public key pair.
  If $a_{i,1}-\frac{a}{\omega }=0$ mod *q*, the above procedure cannot occur and aborts. Otherwise, a private/public key pair is outputted to A.
- ***Atomic-Sign***(): Given the public parameters MRK, the public key list PK_List and the message M, where PK_List is a list of the public keys of the devices involved with this query (with respect to the device *i*), the algorithm finishes the following steps:
  - The algorithm randomly chooses $sk_{i,0},z_{i}\in Z_{q}$ and a vector ($r_{i,k}$) with $r_{i,k}\in Z_{q}$ and k∈[1,2……l], computes $x_{i,0,k}=g^{\frac{sk_{i,0}}{l}}\cdot \varpi^{r_{i,k}}$ and $x_{i,1,k}=g^{r_{i,k}}$ with k∈[1,2……l], and then saves $sk_{i,0}$ where $sk_{i,0}=a_{i,0}$;
  - The message M is divided to $msg_{1},msg_{2},\ldots \ldots msg_{l}$; then the algorithm computes $v_{k}=$ $H(msg_{k}||pk_{k})$ with k∈[1,2……l], where we assume the signing needs to involve *l* devices, $pk_{k}$ is the public key of the *k*-th device with $pk_{k}\in PK\_List$;
  - For $V=(v_{1},v_{2},\ldots \ldots v_{l})$, generate the claim-predicate Υ which satisfies Υ(V)=1, and then transform the claim-predicate Υ to the monotone span program $\Lambda \in {\left(Z_{q}\right)}^{l\times t_{max}}$;
  - Compute $s_{k,j}=\psi^{r_{i,k}}\cdot {\left(u_{j}\right)}^{v_{k}\cdot r_{i,k}}$ with k∈[1,2……l] and $j\in [1,2\ldots \ldots t_{max}]$;
  - The algorithm outputs the atomic signatures $\sigma_{a}=\left\{\left(x_{i,0,k}\right),\left(x_{i,1,k}\right),\left(s_{k,j}\right)\right\}$ to A, where k∈[1,2……l] and $j\in [1,2\ldots \ldots t_{max}]$.
  If $v_{k}=H(msg_{k}||pk_{k})=0$ mod *q* with k∈[1,2……l], then the above procedure cannot ocur and aborts. Otherwise, the atomic signatures are passed to the adversary A.
- ***Mesh-Sign***(): Given the public parameters MRK, the atomic signatures $\sigma_{a}=\left\{\left(x_{i,0,k}\right),\left(x_{i,1,k}\right),\left(s_{k,j}\right)\right\}$ on the public key list PK_List and the message M (with respect to the device *i*), and the monotone boolean expression Υ, the algorithm finishes the following steps:
  - Choose $b,c,t,d_{0},d_{1},\ldots \ldots d_{l},a_{i,1}\in Z_{q}$ randomly, compute $X_{0}=\prod_{k=1}^{l}\left(x_{i,0,k}\right)\cdot \vartheta^{\left(d_{0}+b\right)\cdot H\left(M\right)}\cdot \psi^{d_{0}+b}\cdot \varpi^{d_{0}}$, $X_{1}=g^{d_{0}+b}$, $X_{2}=g^{d_{0}}\cdot \prod_{k=1}^{l}\left(x_{i,1,k}\right)=g^{d_{0}+\sum_{k=1}^{l}r_{i,k}}$, $X_{3}=g^{a_{i,1}}\cdot g_{1}^{-\frac{1}{\omega }}\cdot g^{c}$, $X_{4}=y^{sk_{i,0}}\cdot y^{c}$ according to the corresponding $sk_{i,0}$, $X_{5,k}=x_{i,1,k}\cdot g^{d_{0}+t}=g^{r_{i,k}+d_{0}+t}$ with k∈[1,2……l];
  - Compute the vector $\vec{\eta }=\left(\eta_{k}\right)$ related to the satisfying assignment $V=(v_{1},v_{2},\ldots \ldots v_{l})$, where $\eta_{k}\in Z_{q}$ with k∈[1,2……l];
  - Compute $I_{k}=g^{d_{k}}\cdot {\left(X_{1}\right)}^{\frac{\eta_{k}}{v_{k}}}$ with k∈[1,2……l], $Q_{j}=y^{a_{i,1}}\cdot y^{c}\cdot g^{c}\cdot X_{0}\cdot \prod_{k=1}^{l}\psi^{\left(d_{0}+t\right)\cdot \Lambda_{k,j}}\cdot \prod_{k=1}^{l}\left[{\left(s_{k,j}\right)}^{\Lambda_{k,j}}\cdot {\left(u_{j}\right)}^{\left(d_{0}+t\right)\cdot \Lambda_{k,j}\cdot v_{k}}\cdot {\left(u_{j}\right)}^{d_{k}\cdot \Lambda_{k,j}\cdot v_{k}}\right]$ with j∈[1,2…… $.t_{max}]$;
  - The algorithm finally generates and outputs a mesh signature
    $\sigma_{m}=\left\{X_{1},X_{2},X_{3},X_{4},\left(X_{5,1},\ldots \ldots X_{5,l}\right),\left(I_{1},\ldots \ldots I_{l}\right),\left(Q_{1},\ldots \ldots Q_{t_{max}}\right)\right\}$.
  Similarly, setting $a_{i,1}^{\prime }=a_{i,1}-\frac{a}{\omega }$, $\sigma_{m}$ is a valid mesh signature. If $a_{i,1}-\frac{a}{\omega }=0$ mod *q*, the above procedure cannot occur and aborts. Otherwise, a mesh signature $\sigma_{m}$ is outputted to A.

**Forgery**: If B finally does not abort, then A can return its forgery with probability at least ε, (MRK, $PK\_List^{*}$, $M^{*}$, $\Upsilon^{*}$, $\Phi^{*}$), where $\Upsilon^{*}$ can be converted to the corresponding monotone span program $\Lambda^{*}\in {\left(Z_{q}\right)}^{l\times t_{\max}}$, the vector ${\vec{\eta }}^{*}=\left(\eta_{k}^{*}\right)$ is related to the satisfying assignment $V^{*}=\left(v_{1}^{*},v_{2}^{*},\ldots \ldots v_{l}^{*}\right)$ with k∈[1,2……l]. It succeeds if

(a) accept←***Mesh-Verify***(MRK, $PK\_List^{*}$, $M^{*}$, $\Upsilon^{*}$, $\Phi^{*}$);
(b) A did not query ***Generate-Key*** on any public key belongs to $PK\_List^{*}$, and it did not query ***Mesh-Sign*** on the related inputs $PK\_List^{*}$, $M^{*}$ and $\Upsilon^{*}$.

Then we may get the following:

$$\Phi^{*}=\left\{X_{1}^{*},X_{2}^{*},X_{3}^{*},X_{4}^{*},\left(X_{5,1}^{*},X_{5,2}^{*},\ldots \ldots X_{5,l}^{*}\right),\left({I_{1}}^{*},{I_{2}}^{*},\ldots \ldots {I_{l}}^{*}\right),\left({Q_{1}}^{*},{Q_{2}}^{*},\ldots \ldots Q_{t_{max}}^{*}\right)\right\},$$

where $\Upsilon^{*}\left(V^{*}\right)=1$, and

$X_{1}^{*}=g^{d_{0}^{*}+b^{*}}$,
  $X_{2}^{*}=g^{d_{0}^{*}+\sum_{k=1}^{l}r_{i,k}^{*}}$,
  $X_{3}^{*}=g^{a_{i,1}^{*}+c^{*}}$,
  $X_{4}^{*}=y^{a_{i,0}^{*}+c^{*}}$,
  $X_{5,k}^{*}=g^{r_{i,k}^{*}+d_{0}^{*}+t^{*}}$,
  $I_{k}^{*}=g^{d_{k}^{*}}\cdot {\left(X_{1}^{*}\right)}^{\frac{\eta_{k}^{*}}{v_{k}^{*}}}$,
  $Q_{j}^{*}=f^{a}\cdot y^{a_{i,1}^{*}+c^{*}}\cdot g^{c^{*}+a_{i,0}^{*}}\cdot \vartheta^{\left(d_{0}^{*}+b^{*}\right)\cdot H\left(M^{*}\right)}\cdot \psi^{d_{0}^{*}+b^{*}}\cdot \varpi^{d_{0}^{*}+\sum_{k=1}^{l}r_{i,k}^{*}}\cdot \prod_{k=1}^{l}\psi^{\left(d_{0}^{*}+t+r_{i,k}^{*}\right)\cdot \Lambda_{k,j}^{*}}\cdot \prod_{k=1}^{l}{\left(u_{j}\right)}^{\left(d_{0}^{*}+t^{*}+r_{i,k}^{*}\right)\cdot \Lambda_{k,j}^{*}\cdot v_{k}^{*}}\cdot \prod_{k=1}^{l}{\left(u_{j}\right)}^{d_{k}^{*}\cdot \Lambda_{k,j}^{*}\cdot v_{k}^{*}}$.

Finally, the algorithm B computes and outputs

$$\frac{Q_{j}^{*}}{{\left(X_{3}^{*}\right)}^{\beta }\cdot {\left(X_{4}^{*}\right)}^{\frac{1}{\beta }}\cdot {\left(X_{1}^{*}\right)}^{\iota \cdot H(M^{*})}\cdot {\left(X_{1}^{*}\right)}^{\varphi }\cdot {\left(X_{2}^{*}\right)}^{\varrho }\cdot \prod_{k=1}^{l}{\left(X_{5,k}^{*}\right)}^{\varphi \cdot \Lambda_{k,j}^{*}}\cdot \prod_{k=1}^{l}{\left(X_{5,k}^{*}\right)}^{\ell_{j}\cdot \Lambda_{k,j}^{*}\cdot v_{k}^{*}}\cdot \prod_{k=1}^{l}g^{\ell_{j}\cdot d_{k}^{*}\cdot \Lambda_{k,j}^{*}\cdot v_{k}^{*}}}=f^{a}=g^{a\cdot b},$$

which solves the given CDH problem. Then, we compute the probability that B does not abort. For the complete simulation procedure of B, we must assure that all key queries can have $a_{i,1}-\frac{a}{\omega }\neq 0$ mod *q*, all atomic signature queries can have $v_{k}=H(msg_{k}||pk_{k})\neq 0$ mod *q* for all k∈[1,2……l], and all mesh signature queries can have $a_{i,1}-\frac{a}{\omega }\neq 0$ mod *q*. Therefore, if B will not abort, then we must assure that the following three conditions hold:

(a) $a_{i,1}-\frac{a}{\omega }\neq 0$ mod *q* in related key queries;
(b) $v_{k}=H(msg_{k}||pk_{k})\neq 0$ mod *q* for all k∈[1,2……l] in related atomic signature queries;
(c) $a_{i,1}-\frac{a}{\omega }\neq 0$ mod *q* in related mesh signature queries.

To make our analysis easier to understand, we define the events $E_{j}$, $R_{j}$ and $T_{j}$ as $E_{j}:$$a_{i,1}-\frac{a}{\omega }\neq 0$ mod *q*, with *j* = $1,2\ldots \ldots q_{k}$, $q_{k}$ denotes the queries number of “Generate-Key” oracle; $R_{j}:$$v_{k}=H(msg_{k}||pk_{k})\neq 0$ mod *q* for all k∈[1,2……l], with *j*=$1,2\ldots \ldots q_{a}$, $q_{a}$ denotes the queries number of “Atomic Signature” oracle; $T_{j}:$$a_{i,1}-\frac{a}{\omega }\neq 0$ mod *q*, with *j* = $1,2\ldots \ldots q_{m}$, $q_{m}$ denotes the queries number of “Mesh Signature” oracle. The probability that B is completely simulated is $\mathrm{Pr}\left(not\_abort\right)=\mathrm{Pr}\left(\overset{q_{k}}{\underset{j=1}{⋂}}E_{j}\wedge \overset{q_{a}}{\underset{j=1}{⋂}}R_{j}\wedge \overset{q_{m}}{\underset{j=1}{⋂}}T_{j}\right)$. It is easy to see that the events $\overset{q_{k}}{\underset{j=1}{⋂}}E_{j}$, $\overset{q_{a}}{\underset{j=1}{⋂}}R_{j}$, $\overset{q_{m}}{\underset{j=1}{⋂}}T_{j}$ are independent. Then we may compute

$$\mathrm{Pr}\left(\overset{q_{k}}{\underset{j=1}{⋂}}E_{j}\right)=1-\mathrm{Pr}\left(\overset{q_{k}}{\underset{j=1}{⋃}}\neg E_{j}\right)=1-q_{k}\cdot \frac{1}{q}=1-\frac{q_{k}}{q};$$

$$\mathrm{Pr}\left(\overset{q_{a}}{\underset{j=1}{⋂}}R_{j}\right)=1-\mathrm{Pr}\left(\overset{q_{a}}{\underset{j=1}{⋃}}\neg R_{j}\right)=1-q_{a}\cdot \left[1-{\left(1-\frac{1^{k}}{1^{k}\cdot q}\right)}^{l}\right]=1-q_{a}+q_{a}\cdot {\left(1-\frac{1}{q}\right)}^{l};$$

$$\mathrm{Pr}\left(\overset{q_{m}}{\underset{j=1}{⋂}}T_{j}\right)=1-\mathrm{Pr}\left(\overset{q_{m}}{\underset{j=1}{⋃}}\neg T_{j}\right)=1-q_{m}\cdot \frac{1}{q}=1-\frac{q_{m}}{q}.$$

Therefore,

$$\mathrm{Pr}\left(not\_abort\right)=\mathrm{Pr}\left(\overset{q_{k}}{\underset{j=1}{⋂}}E_{j}\wedge \overset{q_{a}}{\underset{j=1}{⋂}}R_{j}\wedge \overset{q_{m}}{\underset{j=1}{⋂}}T_{j}\right)$$

$$=\mathrm{Pr}\left(\overset{q_{k}}{\underset{j=1}{⋂}}E_{j}\right)\cdot \mathrm{Pr}\left(\overset{q_{a}}{\underset{j=1}{⋂}}R_{j}\right)\cdot \mathrm{Pr}\left(\overset{q_{m}}{\underset{j=1}{⋂}}T_{j}\right)$$

$$=\left(1-\frac{q_{k}}{q}\right)\cdot \left[1-q_{a}+q_{a}\cdot {\left(1-\frac{1}{q}\right)}^{l}\right]\cdot \left(1-\frac{q_{m}}{q}\right).$$

Therefore, we can get that $\varepsilon^{\prime }=\left(1-\frac{q_{k}}{q}\right)\cdot \left[1-q_{a}+q_{a}\cdot {\left(1-\frac{1}{q}\right)}^{l}\right]\cdot \left(1-\frac{q_{m}}{q}\right)\cdot \varepsilon .$ If B is completely simulated, then A generates a valid mesh signature forgery with probability at least ε, and B may be used to compute $g^{a\cdot b}$. The time cost of B mainly includes the time of the exponentiations and multiplications in queries. We assume that the time of other lightweight computations is ignored (such as integer addition, integer multiplication and hash computation), then the time cost of B is

$$\begin{array}{c} \hbar^{\prime }=\hbar +O(q_{k}\cdot \left[3\cdot C_{exp}+C_{mul}\right]+q_{a}\cdot \left[\left(2\cdot l\cdot t_{max}+3\right)\cdot C_{exp}+\left(l\cdot t_{max}+1\right)\cdot C_{mul}\right]+q_{m}\cdot [(4\cdot l\cdot t_{max}+\\ 3\cdot l+13)\cdot C_{exp}+\left(4\cdot l\cdot t_{max}+4\cdot l+8\right)\cdot C_{mul}]). \end{array}$$

Thus, Theorem 1 follows. □

### Appendix A.2. Anonymity

**Proof** **(Proof of Theorem 2).** (This proof is similar to that of Theorem 1, the difference between them is to add the queries of phase 2.) We set that **MS** is our proposed mesh signature scheme. Also we set that A is an adversary with the tuple (*ℏ*, ε, $q_{k}$, $q_{a}$, $q_{m}$) that can make attack to **MS**. To make interaction with the adversary A, an algorithm B is constructed. For (*g*, $g^{a}$, $g^{b}$)$\in G_{1}$, B may make interaction with A to compute $g^{a\cdot b}$. Then the algorithm B can be assumed to solve the CDH problem with probability at least $\varepsilon^{\prime }$ and in time at most $\hbar^{\prime }$, which is contrary to the ($\hbar^{\prime }$, $\varepsilon^{\prime }$)-CDH assumption. Therefore, we may build a simulation procedure as follows: **1. Setup**: The parameter $1^{k}$ is inputted. Also, we set that $G_{1}$ and $G_{2}$ are the groups of prime order *q*, *g* is a generator of $G_{1}$, and that $e:{G_{1}\times G_{1}\to G}_{2}$ denotes the bilinear map. In addition, we set that $H:{\left\{0,1\right\}}^{*}\to Z_{1^{k}\cdot q}$ denotes one hash function and it can be used to output integers in $Z_{1^{k}\cdot q}$. Additionally, we assume that the monotone span programs related to claim-predicates have their width at most $t_{max}$ in our construction. Then the following parameters are outputted. The algorithm sets $g_{1}=g^{a}$ and $f=g^{b}$ with $a,b\in Z_{q}$ (B does not know *a* and *b*), chooses $\omega ,\beta ,\iota ,\varphi ,\varrho \in Z_{q}$, and then sets $y=f^{\omega }=g^{\beta }$, $\vartheta =g^{\iota }$, $\psi =g^{\varphi }$ and $\varpi =g^{\varrho }$. In addition, the algorithm chooses $\ell_{j}\in Z_{q}$ for all *j*s with $j\in [1,2\ldots \ldots t_{max}]$, and then sets $u_{j}=g^{\ell_{j}}$ for all *j*s with $j\in [1,2\ldots \ldots t_{max}]$. Then this algorithm outputs all the parameters MRK=($G_{1}$, $G_{2}$, *e*, *g*, $g_{1}$, *y*, *f*, ϑ, ψ, ϖ, $\Psi =(u_{j})$), where msk=a is seen as the master key of the system. **2. Queries Phase 1**: A makes the following key and signature queries, then B gives its answers as follows:

- ***Generate-Key***(): Given the public parameters MRK, for the device *i*, the algorithm randomly chooses $a_{i,0},a_{i,1}\in Z_{q}$, sets $sk_{i,0}=a_{i,0}$ and computes $sk_{i,1}=y^{a_{i,1}}$, $pk_{i,0}=g^{a_{i,0}}$ and $pk_{i,1}=g^{a_{i,1}}\cdot g_{1}^{-\frac{1}{\omega }}$, where $sk_{i}$ = ($sk_{i,0}$, $sk_{i,1}$) is the private key of the device *i* and $pk_{i}$ = ($pk_{i,0}$, $pk_{i,1}$) is the public key of the device *i*, and then the private/public key pair is passed to the adversary A. Similarly, setting $a_{i,1}^{\prime }=a_{i,1}-\frac{a}{\omega }$, then $sk_{i,1}=f^{a}\cdot y^{a_{i,1}^{\prime }}$ and $pk_{i,1}=g^{a_{i,1}^{\prime }}$. Therefore, $sk_{i}$ and $pk_{i}$ is a valid private/public key pair.
  If $a_{i,1}-\frac{a}{\omega }=0$ mod *q*, the above procedure cannot occur and aborts. Otherwise, a private/public key pair is outputted to A.
- ***Atomic-Sign***(): Given the public parameters MRK, the public key list PK_List and the message M, where PK_List is a list of the public keys of the devices involved with this query (with respect to the device *i*), the algorithm finishes the following steps:
  - The algorithm randomly chooses $sk_{i,0},z_{i}\in Z_{q}$ and a vector ($r_{i,k}$) with $r_{i,k}\in Z_{q}$ and k∈[1,2……l], computes $x_{i,0,k}=g^{\frac{sk_{i,0}}{l}}\cdot \varpi^{r_{i,k}}$ and $x_{i,1,k}=g^{r_{i,k}}$ with k∈[1,2……l], and then saves $sk_{i,0}$ where $sk_{i,0}=a_{i,0}$;
  - The message M is divided to $msg_{1},msg_{2},\ldots \ldots msg_{l}$; then the algorithm computes $v_{k}=H(msg_{k}||pk_{k})$ with k∈[1,2……l], where we assume the signing needs to involve *l* devices, $pk_{k}$ is the public key of the *k*-th device with $pk_{k}\in PK\_List$;
  - For $V=(v_{1},v_{2},\ldots \ldots v_{l})$, generate the claim-predicate Υ which satisfies Υ(V)=1, and then transform the claim-predicate Υ to the monotone span program $\Lambda \in {\left(Z_{q}\right)}^{l\times t_{max}}$;
  - Compute $s_{k,j}=\psi^{r_{i,k}}\cdot {\left(u_{j}\right)}^{v_{k}\cdot r_{i,k}}$ with k∈[1,2……l] and $j\in [1,2\ldots \ldots t_{max}]$;
  - The algorithm outputs the atomic signatures $\sigma_{a}=\left\{\left(x_{i,0,k}\right),\left(x_{i,1,k}\right),\left(s_{k,j}\right)\right\}$ to A, where k∈[1,2……l] and $j\in [1,2\ldots \ldots t_{max}]$.
  If $v_{k}=H(msg_{k}||pk_{k})=0$ mod *q* with k∈[1,2……l], then the above procedure cannot occur and will abort; otherwise the atomic signatures are passed to the adversary A.
- ***Mesh-Sign***(): Given the public parameters MRK, the atomic signatures $\sigma_{a}=\left\{\left(x_{i,0,k}\right),\left(x_{i,1,k}\right),\left(s_{k,j}\right)\right\}$ on the public key list PK_List and the message M (with respect to the device *i*), and the monotone boolean expression Υ, the algorithm finishes the following steps:
  - Choose $b,c,t,d_{0},d_{1},\ldots \ldots d_{l},a_{i,1}\in Z_{q}$ randomly, compute $X_{0}=\prod_{k=1}^{l}\left(x_{i,0,k}\right)\cdot \vartheta^{\left(d_{0}+b\right)\cdot H\left(M\right)}\cdot \psi^{d_{0}+b}\cdot \varpi^{d_{0}}$, $X_{1}=g^{d_{0}+b}$, $X_{2}=g^{d_{0}}\cdot \prod_{k=1}^{l}\left(x_{i,1,k}\right)=g^{d_{0}+\sum_{k=1}^{l}r_{i,k}}$, $X_{3}=g^{a_{i,1}}\cdot g_{1}^{-\frac{1}{\omega }}\cdot g^{c}$, $X_{4}=y^{sk_{i,0}}\cdot y^{c}$ according to the corresponding $sk_{i,0}$, $X_{5,k}=x_{i,1,k}\cdot g^{d_{0}+t}=g^{r_{i,k}+d_{0}+t}$ with k∈[1,2……l];
  - Compute the vector $\vec{\eta }=\left(\eta_{k}\right)$ related to the satisfying assignment $V=(v_{1},v_{2},\ldots \ldots v_{l})$, where $\eta_{k}\in Z_{q}$ with k∈[1,2……l];
  - Compute $I_{k}=g^{d_{k}}\cdot {\left(X_{1}\right)}^{\frac{\eta_{k}}{v_{k}}}$ with k∈[1,2……l], $Q_{j}=y^{a_{i,1}}\cdot y^{c}\cdot g^{c}\cdot X_{0}\cdot \prod_{k=1}^{l}\psi^{\left(d_{0}+t\right)\cdot \Lambda_{k,j}}\cdot \prod_{k=1}^{l}\left[{\left(s_{k,j}\right)}^{\Lambda_{k,j}}\cdot {\left(u_{j}\right)}^{\left(d_{0}+t\right)\cdot \Lambda_{k,j}\cdot v_{k}}\cdot {\left(u_{j}\right)}^{d_{k}\cdot \Lambda_{k,j}\cdot v_{k}}\right]$ with j∈[1,2…… $.t_{max}]$;
  - The algorithm finally generates and outputs a mesh signature
    $\sigma_{m}=\left\{X_{1},X_{2},X_{3},X_{4},\left(X_{5,1},\ldots \ldots X_{5,l}\right),\left(I_{1},\ldots \ldots I_{l}\right),\left(Q_{1},\ldots \ldots Q_{t_{max}}\right)\right\}$.
  Similarly, setting $a_{i,1}^{\prime }=a_{i,1}-\frac{a}{\omega }$, $\sigma_{m}$ is a valid mesh signature. If $a_{i,1}-\frac{a}{\omega }=0$ mod *q*, the above procedure cannot occur and aborts. Otherwise, a mesh signature $\sigma_{m}$ is passed to A.

**3. Challenge**: The adversary A sends its forgeries (MRK, $PK\_List^{*}\cup \left\{pk_{0}^{*}\right\}\cup \left\{pk_{1}^{*}\right\}$, $M^{*}$, $\Upsilon^{*}$, $\Phi^{*}$) to the challenger. The following conditions are satisfies:

(a) The adversary did not make query to ***Generate-Key*** on $pk_{0}^{*}$ (and $pk_{1}^{*}$);
(b) The adversary did not make query to ***Atomic-Sign*** on $pk_{0}^{*}$ (and $pk_{1}^{*}$);
(c) The adversary did not make query to ***Mesh-Sign*** on $pk_{0}^{*}$ (and $pk_{1}^{*}$).

The challenger randomly chooses a bit x∈{0,1}, and then the following is outputted as $\sigma^{*}\leftarrow$Mesh-Sign (MRK, $sk_{x}^{*}$, $PK\_List^{*}\cup \left\{pk_{0}^{*}\right\}\cup \left\{pk_{1}^{*}\right\}$, M) to A. **4. Queries Phase 2**: A makes the following key and signature queries, then B gives its answers as follows:

- ***Generate-Key***(): Given the public parameters MRK, for the device *i*, the algorithm randomly chooses $a_{i,0},a_{i,1}\in Z_{q}$, sets $sk_{i,0}=a_{i,0}$ and computes $sk_{i,1}=y^{a_{i,1}}$, $pk_{i,0}=g^{a_{i,0}}$ and $pk_{i,1}=g^{a_{i,1}}\cdot g_{1}^{-\frac{1}{\omega }}$, where $sk_{i}$ = ($sk_{i,0}$, $sk_{i,1}$) is the private key of the device *i* and $pk_{i}$ = ($pk_{i,0}$, $pk_{i,1}$) is the public key of the device *i*, and then the private/public key pair is passed to the adversary A. Similarly, setting $a_{i,1}^{\prime }=a_{i,1}-\frac{a}{\omega }$, then $sk_{i,1}=f^{a}\cdot y^{a_{i,1}^{\prime }}$ and $pk_{i,1}=g^{a_{i,1}^{\prime }}$. Therefore, $sk_{i}$ and $pk_{i}$ is a valid private/public key pair.
  If $a_{i,1}-\frac{a}{\omega }=0$ mod *q*, the above procedure cannot occur and aborts. Otherwise, a private/public key pair is outputted to A.
- ***Atomic-Sign***(): Given the public parameters MRK, the public key list PK_List and the message M, where PK_List is a list of the public keys of the devices involved with this query (with respect to the device *i*), the algorithm finishes the following steps:
  - The algorithm randomly chooses $sk_{i,0},z_{i}\in Z_{q}$ and a vector ($r_{i,k}$) with $r_{i,k}\in Z_{q}$ and k∈[1,2……,l], computes $x_{i,0,k}=g^{\frac{sk_{i,0}}{l}}\cdot \varpi^{r_{i,k}}$ and $x_{i,1,k}=g^{r_{i,k}}$ with k∈[1,2……,l], and then saves $sk_{i,0}$ where $sk_{i,0}=a_{i,0}$;
  - The message M is divided to $msg_{1},msg_{2},\ldots \ldots ,msg_{l}$; then the algorithm computes $v_{k}=H(msg_{k}||pk_{k})$ with k∈[1,2……,l], where we assume the signing needs to involve *l* devices, $pk_{k}$ is the public key of the *k*-th device with $pk_{k}\in PK\_List$;
  - For $V=(v_{1},v_{2},\ldots \ldots ,v_{l})$, generate the claim-predicate Υ which satisfies Υ(V)=1, and then transform the claim-predicate Υ to the monotone span program $\Lambda \in {\left(Z_{q}\right)}^{l\times t_{max}}$;
  - Compute $s_{k,j}=\psi^{r_{i,k}}\cdot {\left(u_{j}\right)}^{v_{k}\cdot r_{i,k}}$ with k∈[1,2……,l] and $j\in [1,2\ldots \ldots ,t_{max}]$;
  - The algorithm outputs the atomic signatures $\sigma_{a}=\left\{\left(x_{i,0,k}\right),\left(x_{i,1,k}\right),\left(s_{k,j}\right)\right\}$ to A, where k∈[1,2……,l] and $j\in [1,2\ldots \ldots ,t_{max}]$.
  If $v_{k}=H(msg_{k}||pk_{k})=0$ mod *q* with k∈[1,2……,l], the above procedure cannot be occur and will abort; otherwise the atomic signatures are passed to the adversary A.
- ***Mesh-Sign***(): Given the public parameters MRK, the atomic signatures $\sigma_{a}=\left\{\left(x_{i,0,k}\right),\left(x_{i,1,k}\right),\left(s_{k,j}\right)\right\}$ on the public key list PK_List and the message M (with respect to the device *i*), and the monotone boolean expression Υ, the algorithm finishes the following steps:
  - Choose $b,c,t,d_{0},d_{1},\ldots \ldots ,d_{l},a_{i,1}\in Z_{q}$ randomly, compute $X_{0}=\prod_{k=1}^{l}\left(x_{i,0,k}\right)\cdot \vartheta^{\left(d_{0}+b\right)\cdot H\left(M\right)}\cdot \psi^{d_{0}+b}\cdot \varpi^{d_{0}}$, $X_{1}=g^{d_{0}+b}$, $X_{2}=g^{d_{0}}\cdot \prod_{k=1}^{l}\left(x_{i,1,k}\right)=g^{d_{0}+\sum_{k=1}^{l}r_{i,k}}$, $X_{3}=g^{a_{i,1}}\cdot g_{1}^{-\frac{1}{\omega }}\cdot g^{c}$, $X_{4}=y^{sk_{i,0}}\cdot y^{c}$ according to the corresponding $sk_{i,0}$, $X_{5,k}=x_{i,1,k}\cdot g^{d_{0}+t}=g^{r_{i,k}+d_{0}+t}$ with k∈[1,2……,l];
  - Compute the vector $\vec{\eta }=\left(\eta_{k}\right)$ related to the satisfying assignment $V=(v_{1},v_{2},\ldots \ldots ,v_{l})$, where $\eta_{k}\in Z_{q}$ with k∈[1,2……,l];
  - Compute $I_{k}=g^{d_{k}}\cdot {\left(X_{1}\right)}^{\frac{\eta_{k}}{v_{k}}}$ with k∈[1,2……,l], $Q_{j}=y^{a_{i,1}}\cdot y^{c}\cdot g^{c}\cdot X_{0}\cdot \prod_{k=1}^{l}\psi^{\left(d_{0}+t\right)\cdot \Lambda_{k,j}}\cdot \prod_{k=1}^{l}\left[{\left(s_{k,j}\right)}^{\Lambda_{k,j}}\cdot {\left(u_{j}\right)}^{\left(d_{0}+t\right)\cdot \Lambda_{k,j}\cdot v_{k}}\cdot {\left(u_{j}\right)}^{d_{k}\cdot \Lambda_{k,j}\cdot v_{k}}\right]$ with j∈[1,2……, $.t_{max}]$;
  - The algorithm finally generates and outputs a mesh signature
    $\sigma_{m}=\left\{X_{1},X_{2},X_{3},X_{4},\left(X_{5,1},\ldots \ldots ,X_{5,l}\right),\left(I_{1},\ldots \ldots ,I_{l}\right),\left(Q_{1},\ldots \ldots ,Q_{t_{max}}\right)\right\}.$
  Similarly, setting $a_{i,1}^{\prime }=a_{i,1}-\frac{a}{\omega }$, $\sigma_{m}$ is a valid mesh signature. If $a_{i,1}-\frac{a}{\omega }=0$ mod *q*, the above procedure cannot occur and aborts. Otherwise, a mesh signature $\sigma_{m}$ is passed to A.

**5. Guess**: If B finally does not abort, then the adversary A can output its result $x^{\prime }\in \left\{0,1\right\}$ with probability at least ε and succeeds if $x^{\prime }=x$. Then we may get the following:

$$\Phi^{*}=\left\{X_{1}^{*},X_{2}^{*},X_{3}^{*},X_{4}^{*},\left(X_{5,1}^{*},X_{5,2}^{*},\ldots \ldots ,X_{5,l}^{*}\right),\left({I_{1}}^{*},{I_{2}}^{*},\ldots \ldots ,{I_{l}}^{*}\right),\left({Q_{1}}^{*},{Q_{2}}^{*},\ldots \ldots ,Q_{t_{max}}^{*}\right)\right\},$$

where $\Upsilon^{*}\left(V^{*}\right)=1$, and

$X_{1}^{*}=g^{d_{0}^{*}+b^{*}}$,
  $X_{2}^{*}=g^{d_{0}^{*}+\sum_{k=1}^{l}r_{i,k}^{*}}$,
  $X_{3}^{*}=g^{a_{i,1}^{*}+c^{*}}$,
  $X_{4}^{*}=y^{a_{i,0}^{*}+c^{*}}$,
  $X_{5,k}^{*}=g^{r_{i,k}^{*}+d_{0}^{*}+t^{*}}$,
  $I_{k}^{*}=g^{d_{k}^{*}}\cdot {\left(X_{1}^{*}\right)}^{\frac{\eta_{k}^{*}}{v_{k}^{*}}}$,
  $Q_{j}^{*}=f^{a}\cdot y^{a_{i,1}^{*}+c^{*}}\cdot g^{c^{*}+a_{i,0}^{*}}\cdot \vartheta^{\left(d_{0}^{*}+b^{*}\right)\cdot H\left(M^{*}\right)}\cdot \psi^{d_{0}^{*}+b^{*}}\cdot \varpi^{d_{0}^{*}+\sum_{k=1}^{l}r_{i,k}^{*}}\cdot \prod_{k=1}^{l}\psi^{\left(d_{0}^{*}+t+r_{i,k}^{*}\right)\cdot \Lambda_{k,j}^{*}}\cdot \prod_{k=1}^{l}{\left(u_{j}\right)}^{\left(d_{0}^{*}+t^{*}+r_{i,k}^{*}\right)\cdot \Lambda_{k,j}^{*}\cdot v_{k}^{*}}\cdot \prod_{k=1}^{l}{\left(u_{j}\right)}^{d_{k}^{*}\cdot \Lambda_{k,j}^{*}\cdot v_{k}^{*}}$.

Therefore, the algorithm B computes and outputs

$$\frac{Q_{j}^{*}}{{\left(X_{3}^{*}\right)}^{\beta }\cdot {\left(X_{4}^{*}\right)}^{\frac{1}{\beta }}\cdot {\left(X_{1}^{*}\right)}^{\iota \cdot H(M^{*})}\cdot {\left(X_{1}^{*}\right)}^{\varphi }\cdot {\left(X_{2}^{*}\right)}^{\varrho }\cdot \prod_{k=1}^{l}{\left(X_{5,k}^{*}\right)}^{\varphi \cdot \Lambda_{k,j}^{*}}\cdot \prod_{k=1}^{l}{\left(X_{5,k}^{*}\right)}^{\ell_{j}\cdot \Lambda_{k,j}^{*}\cdot v_{k}^{*}}\cdot \prod_{k=1}^{l}g^{\ell_{j}\cdot d_{k}^{*}\cdot \Lambda_{k,j}^{*}\cdot v_{k}^{*}}}=f^{a}=g^{a\cdot b},$$

which solves the given CDH problem. Then we compute the probability that B does not abort. For the complete simulation procedure of B, we must assure that all key queries can have $a_{i,1}-\frac{a}{\omega }\neq 0$ mod *q* in related queries Phases 1 and 2, all atomic signature queries can have $v_{k}=H(msg_{k}||pk_{k})\neq 0$ mod *q* for all k∈[1,2,……,l] in related Queries Phases 1 and 2, and all mesh signature queries can have $a_{i,1}-\frac{a}{\omega }\neq 0$ mod *q* in related Queries Phases 1 and 2. Therefore, if B will not abort, then we must assure that the following three conditions hold:

(a) $a_{i,1}-\frac{a}{\omega }\neq 0$ mod *q* in the related key queries of Queries Phases 1 and 2;
(b) $v_{k}=H(msg_{k}||pk_{k})\neq 0$ mod *q* for all k∈[1,2,……,l] in the related atomic signature queries of Queries Phases 1 and 2;
(c) $a_{i,1}-\frac{a}{\omega }\neq 0$ mod *q* in the related mesh signature queries of Queries Phases 1 and 2.

To make our analysis easier to understand, we define the following events $E_{j_{1}}$, $R_{j_{1}}$, $T_{j_{1}}$, $E_{j_{2}}$, $R_{j_{2}}$ and $T_{j_{2}}$ as $E_{j_{1}}:$$a_{i,1}-\frac{a}{\omega }\neq 0$ mod *q*, with $j_{1}$ = $1,2,\ldots \ldots ,q_{k_{1}}$, $q_{k_{1}}$ denotes the queries number of “Generate-Key” oracle in related Queries Phase 1; $R_{j_{1}}:$$v_{k}=H(msg_{k}||pk_{k})\neq 0$ mod *q* for all k∈[1,2,……,l], with $j_{1}$=$1,2,\ldots \ldots ,q_{a_{1}}$, $q_{a_{1}}$ denotes the queries number of “Atomic Signature” oracle in related Queries Phase 1; $T_{j_{1}}:$$a_{i,1}-\frac{a}{\omega }\neq 0$ mod *q*, with $j_{1}$ = $1,2,\ldots \ldots ,q_{m_{1}}$, $q_{m_{1}}$ denotes the queries number of “Mesh Signature” oracle in related Queries Phase 1; $E_{j_{2}}:$$a_{i,1}-\frac{a}{\omega }\neq 0$ mod *q*, with $j_{2}$ = $1,2,\ldots \ldots ,q_{k_{2}}$, $q_{k_{2}}$ denotes the queries number of “Generate-Key” oracle in related Queries Phase 2; $R_{j_{2}}:$$v_{k}=H(msg_{k}||pk_{k})\neq 0$ mod *q* for all k∈[1,2,……,l], with $j_{2}$ = $1,2,\ldots \ldots ,q_{a_{2}}$, $q_{a_{2}}$ denotes the queries number of “Atomic Signature” oracle in related Queries Phase 2; $T_{j_{2}}:$$a_{i,1}-\frac{a}{\omega }\neq 0$ mod *q*, with $j_{2}$ = $1,2,\ldots \ldots ,q_{m_{2}}$, $q_{m_{2}}$ denotes the queries number of “Mesh Signature” oracle in related Queries Phase 2. Therefore, the probability that B is completely simulated is $\mathrm{Pr}\left(not\_abort\right)=\mathrm{Pr}\left(\overset{q_{k_{1}}}{\underset{j_{1}=1}{⋂}}E_{j_{1}}\wedge \overset{q_{a_{1}}}{\underset{j_{1}=1}{⋂}}R_{j_{1}}\wedge \overset{q_{m_{1}}}{\underset{j_{1}=1}{⋂}}T_{j_{1}}\wedge \overset{q_{k_{2}}}{\underset{j_{2}=1}{⋂}}E_{j_{2}}\wedge \overset{q_{a_{2}}}{\underset{j_{2}=1}{⋂}}R_{j_{2}}\wedge \overset{q_{m_{2}}}{\underset{j_{2}=1}{⋂}}T_{j_{2}}\right)$. It is easy to see that the events $\overset{q_{k_{1}}}{\underset{j_{1}=1}{⋂}}E_{j_{1}}$, $\overset{q_{a_{1}}}{\underset{j_{1}=1}{⋂}}R_{j_{1}}$, $\overset{q_{m_{1}}}{\underset{j_{1}=1}{⋂}}T_{j_{1}}$, $\overset{q_{k_{2}}}{\underset{j_{2}=1}{⋂}}E_{j_{2}}$, $\overset{q_{a_{2}}}{\underset{j_{2}=1}{⋂}}R_{j_{2}}$ and $\overset{q_{m_{2}}}{\underset{j_{2}=1}{⋂}}T_{j_{2}}$ are independent. Then we may compute

$$\mathrm{Pr}\left(\overset{q_{k_{1}}}{\underset{j_{1}=1}{⋂}}E_{j_{1}}\right)=1-\mathrm{Pr}\left(\overset{q_{k_{1}}}{\underset{j_{1}=1}{⋃}}\neg E_{j_{1}}\right)=1-q_{k_{1}}\cdot \frac{1}{q}=1-\frac{q_{k_{1}}}{q};$$

$$\mathrm{Pr}\left(\overset{q_{a_{1}}}{\underset{j_{1}=1}{⋂}}R_{j_{1}}\right)=1-\mathrm{Pr}\left(\overset{q_{a_{1}}}{\underset{j_{1}=1}{⋃}}\neg R_{j_{1}}\right)=1-q_{a_{1}}\cdot \left[1-{\left(1-\frac{1^{k}}{1^{k}\cdot q}\right)}^{l}\right]=1-q_{a_{1}}+q_{a_{1}}\cdot {\left(1-\frac{1}{q}\right)}^{l};$$

$$\mathrm{Pr}\left(\overset{q_{m_{1}}}{\underset{j_{1}=1}{⋂}}T_{j_{1}}\right)=1-\mathrm{Pr}\left(\overset{q_{m_{1}}}{\underset{j_{1}=1}{⋃}}\neg T_{j_{1}}\right)=1-q_{m_{1}}\cdot \frac{1}{q}=1-\frac{q_{m_{1}}}{q};$$

$$\mathrm{Pr}\left(\overset{q_{k_{2}}}{\underset{j_{2}=1}{⋂}}E_{j_{2}}\right)=1-\mathrm{Pr}\left(\overset{q_{k_{2}}}{\underset{j_{2}=1}{⋃}}\neg E_{j_{2}}\right)=1-q_{k_{2}}\cdot \frac{1}{q}=1-\frac{q_{k_{2}}}{q};$$

$$\mathrm{Pr}\left(\overset{q_{a_{2}}}{\underset{j_{2}=1}{⋂}}R_{j_{2}}\right)=1-\mathrm{Pr}\left(\overset{q_{a_{2}}}{\underset{j_{2}=1}{⋃}}\neg R_{j_{2}}\right)=1-q_{a_{2}}\cdot \left[1-{\left(1-\frac{1^{k}}{1^{k}\cdot q}\right)}^{l}\right]=1-q_{a_{2}}+q_{a_{2}}\cdot {\left(1-\frac{1}{q}\right)}^{l};$$

$$\mathrm{Pr}\left(\overset{q_{m_{2}}}{\underset{j_{2}=1}{⋂}}T_{j_{2}}\right)=1-\mathrm{Pr}\left(\overset{q_{m_{2}}}{\underset{j_{2}=1}{⋃}}\neg T_{j_{2}}\right)=1-q_{m_{2}}\cdot \frac{1}{q}=1-\frac{q_{m_{2}}}{q}.$$

Therefore,

$$\mathrm{Pr}\left(not\_abort\right)=\mathrm{Pr}\left(\overset{q_{k_{1}}}{\underset{j_{1}=1}{⋂}}E_{j_{1}}\wedge \overset{q_{a_{1}}}{\underset{j_{1}=1}{⋂}}R_{j_{1}}\wedge \overset{q_{m_{1}}}{\underset{j_{1}=1}{⋂}}T_{j_{1}}\wedge \overset{q_{k_{2}}}{\underset{j_{2}=1}{⋂}}E_{j_{2}}\wedge \overset{q_{a_{2}}}{\underset{j_{2}=1}{⋂}}R_{j_{2}}\wedge \overset{q_{m_{2}}}{\underset{j_{2}=1}{⋂}}T_{j_{2}}\right)$$

$$=\left(1-\frac{q_{k_{1}}}{q}\right)\cdot \left(1-\frac{q_{k_{2}}}{q}\right)\cdot \left[1-q_{a_{1}}+q_{a_{1}}\cdot {\left(1-\frac{1}{q}\right)}^{l}\right]\cdot \left[1-q_{a_{2}}+q_{a_{2}}\cdot {\left(1-\frac{1}{q}\right)}^{l}\right]\cdot \left(1-\frac{q_{m_{1}}}{q}\right)\cdot \left(1-\frac{q_{m_{2}}}{q}\right).$$

Therefore, we can get that $\varepsilon^{\prime }=\left(1-\frac{q_{k_{1}}}{q}\right)\cdot \left(1-\frac{q_{k_{2}}}{q}\right)\cdot \left[1-q_{a_{1}}+q_{a_{1}}\cdot {\left(1-\frac{1}{q}\right)}^{l}\right]\cdot \left[1-q_{a_{2}}+q_{a_{2}}\cdot {\left(1-\frac{1}{q}\right)}^{l}\right]\cdot \left(1-\frac{q_{m_{1}}}{q}\right)\cdot \left(1-\frac{q_{m_{2}}}{q}\right)\cdot \left(\varepsilon -\frac{1}{2}\right).$ If B is completely simulated, then A generates a valid mesh signature forgery with probability at least ε, and B may be used to compute $g^{a\cdot b}$. The time cost of B mainly includes the time of the exponentiations and multiplications in queries. We assume that the time of other lightweight computations is ignored, then the time cost of B is

$$\hbar^{\prime }=\hbar +O\left(\left(q_{k_{1}}+q_{k_{2}}\right)\cdot \left[3\cdot C_{exp}+C_{mul}\right]+\left(q_{a_{1}}+q_{a_{2}}\right)\cdot \left[\left(2\cdot l\cdot t_{max}+3\right)\cdot C_{exp}+\left(l\cdot t_{max}+1\right)\cdot C_{mul}\right]+\left(q_{m_{1}}+q_{m_{2}}\right)\cdot \left[\left(4\cdot l\cdot t_{max}+3\cdot l+13\right)\cdot C_{exp}+\left(4\cdot l\cdot t_{max}+4\cdot l+8\right)\cdot C_{mul}\right]\right).$$

Thus, Theorem 2 follows. □

### Appendix A.3. Correctness

In the proposed scheme, the mesh signature is

$$\Phi =\{X_{1},X_{2},X_{3},X_{4},\left(X_{5,1},\ldots \ldots ,X_{5,l}\right),\left(I_{1},\ldots \ldots ,I_{l}\right),\left(Q_{1},\ldots \ldots ,Q_{t_{max}}\right)\},$$

where

$X_{0}=\prod_{k=1}^{l}\left(x_{i,0,k}\right)\cdot \vartheta^{\left(d_{0}+b\right)\cdot H\left(M\right)}\cdot \psi^{d_{0}+b}\cdot \varpi^{d_{0}}$
  $=\prod_{k=1}^{l}\left(g^{\frac{sk_{i,0}}{l}}\cdot \varpi^{r_{i,k}}\right)\cdot \vartheta^{\left(d_{0}+b\right)\cdot H\left(M\right)}\cdot \psi^{d_{0}+b}\cdot \varpi^{d_{0}}$
  $=g^{sk_{i,0}}\cdot \varpi^{\sum_{k=1}^{l}r_{i,k}}\cdot \vartheta^{\left(d_{0}+b\right)\cdot H\left(M\right)}\cdot \psi^{d_{0}+b}\cdot \varpi^{d_{0}}$
  $=g^{a_{i,0}}\cdot \vartheta^{\left(d_{0}+b\right)\cdot H\left(M\right)}\cdot \psi^{d_{0}+b}\cdot \varpi^{d_{0}+\sum_{k=1}^{l}r_{i,k}}$,
  $X_{1}=g^{d_{0}+b}$,
  $X_{2}=g^{d_{0}}\cdot \prod_{k=1}^{l}\left(x_{i,1,k}\right)=g^{d_{0}+\sum_{k=1}^{l}r_{i,k}}$,
  $X_{3}=pk_{i,1}\cdot g^{c}$,
  $X_{4}=y^{a_{i,0}}\cdot y^{c}$,
  $X_{5,k}=x_{i,1,k}\cdot g^{d_{0}+t}=g^{r_{i,k}+d_{0}+t}$,
  $I_{k}=g^{d_{k}}\cdot {\left(X_{1}\right)}^{\frac{\eta_{k}}{v_{k}}}$,
  $Q_{j}=sk_{i,1}\cdot y^{c}\cdot g^{c}\cdot X_{0}\cdot \prod_{k=1}^{l}\psi^{\left(d_{0}+t\right)\cdot \Lambda_{k,j}}\cdot \prod_{k=1}^{l}\left[{\left(s_{k,j}\right)}^{\Lambda_{k,j}}\cdot {\left(u_{j}\right)}^{\left(d_{0}+t\right)\cdot \Lambda_{k,j}\cdot v_{k}}\cdot {\left(u_{j}\right)}^{d_{k}\cdot \Lambda_{k,j}\cdot v_{k}}\right]$
  $=f^{a}\cdot y^{a_{i,1}}\cdot y^{c}\cdot g^{c}\cdot g^{a_{i,0}}\cdot \vartheta^{\left(d_{0}+b\right)\cdot H\left(M\right)}\cdot \psi^{d_{0}+b}\cdot \varpi^{d_{0}+\sum_{k=1}^{l}r_{i,k}}\cdot \prod_{k=1}^{l}\psi^{\left(d_{0}+t\right)\cdot \Lambda_{k,j}}\cdot \prod_{k=1}^{l}\left[{\left(s_{k,j}\right)}^{\Lambda_{k,j}}\cdot {\left(u_{j}\right)}^{\left(d_{0}+t\right)\cdot \Lambda_{k,j}\cdot v_{k}}\cdot {\left(u_{j}\right)}^{d_{k}\cdot \Lambda_{k,j}\cdot v_{k}}\right]$
  $=f^{a}\cdot y^{a_{i,1}+c}\cdot g^{c+a_{i,0}}\cdot \vartheta^{\left(d_{0}+b\right)\cdot H\left(M\right)}\cdot \psi^{d_{0}+b}\cdot \varpi^{d_{0}+\sum_{k=1}^{l}r_{i,k}}\cdot \prod_{k=1}^{l}\psi^{\left(d_{0}+t\right)\cdot \Lambda_{k,j}}\cdot \prod_{k=1}^{l}\left[{\left(\psi^{r_{i,k}}\cdot \left(u_{j}^{v_{k}\cdot r_{i,k}}\right)\right)}^{\Lambda_{k,j}}\cdot {\left(u_{j}\right)}^{\left(d_{0}+t\right)\cdot \Lambda_{k,j}\cdot v_{k}}\cdot {\left(u_{j}\right)}^{d_{k}\cdot \Lambda_{k,j}\cdot v_{k}}\right]$
  $=f^{a}\cdot y^{a_{i,1}+c}\cdot g^{c+a_{i,0}}\cdot \vartheta^{\left(d_{0}+b\right)\cdot H\left(M\right)}\cdot \psi^{d_{0}+b}\cdot \varpi^{d_{0}+\sum_{k=1}^{l}r_{i,k}}\cdot \prod_{k=1}^{l}\psi^{\left(d_{0}+t\right)\cdot \Lambda_{k,j}}\cdot \prod_{k=1}^{l}\psi^{r_{i,k}\cdot \Lambda_{k,j}}\cdot \prod_{k=1}^{l}{\left(u_{j}\right)}^{v_{k}\cdot r_{i,k}\cdot \Lambda_{k,j}}\cdot \prod_{k=1}^{l}{\left(u_{j}\right)}^{\left(d_{0}+t\right)\cdot \Lambda_{k,j}\cdot v_{k}}\cdot \prod_{k=1}^{l}{\left(u_{j}\right)}^{d_{k}\cdot \Lambda_{k,j}\cdot v_{k}}$
  $=f^{a}\cdot y^{a_{i,1}+c}\cdot g^{c+a_{i,0}}\cdot \vartheta^{\left(d_{0}+b\right)\cdot H\left(M\right)}\cdot \psi^{d_{0}+b}\cdot \varpi^{d_{0}+\sum_{k=1}^{l}r_{i,k}}\cdot \prod_{k=1}^{l}\psi^{\left(d_{0}+t+r_{i,k}\right)\cdot \Lambda_{k,j}}\cdot \prod_{k=1}^{l}{\left(u_{j}\right)}^{\left(d_{0}+t+r_{i,k}\right)\cdot \Lambda_{k,j}\cdot v_{k}}\cdot \prod_{k=1}^{l}{\left(u_{j}\right)}^{d_{k}\cdot \Lambda_{k,j}\cdot v_{k}}$.

Therefore, we have that $e\left(f,g_{1}\right)\cdot e\left(y,X_{3}\right)\cdot e\left(g,X_{4}\right)\cdot e\left(\vartheta^{H(M)}\cdot \psi ,X_{1}\right)\cdot e\left(\varpi ,X_{2}\right)\cdot \prod_{k=1}^{l}e\left(\psi^{\Lambda_{k,j}}\cdot {u_{j}}^{v_{k}\cdot \Lambda_{k,j}},X_{5,k}\right)\cdot \prod_{k=1}^{l}e\left({u_{j}}^{v_{k}\cdot \Lambda_{k,j}},I_{k}\right)$

$=e\left(f,g^{a}\right)\cdot e\left(y,g^{a_{i,1}+c}\right)\cdot e\left(g,y^{c+a_{i,0}}\right)\cdot e\left(\vartheta^{H(M)}\cdot \psi ,g^{d_{0}+b}\right)\cdot e\left(\varpi ,g^{d_{0}+\sum_{k=1}^{l}r_{i,k}}\right)\cdot \prod_{k=1}^{l}e\left(\psi^{\Lambda_{k,j}}\cdot {u_{j}}^{v_{k}\cdot \Lambda_{k,j}},g^{r_{i,k}+d_{0}+t}\right)\cdot \prod_{k=1}^{l}e\left({u_{j}}^{v_{k}\cdot \Lambda_{k,j}},g^{d_{k}}\cdot {\left(X_{1}\right)}^{\frac{\eta_{k}}{v_{k}}}\right)$

$=e\left(f^{a}\cdot y^{a_{i,1}+c}\cdot g^{c+a_{i,0}}\cdot \vartheta^{\left(d_{0}+b\right)\cdot H\left(M\right)}\cdot \psi^{d_{0}+b}\cdot \varpi^{d_{0}+\sum_{k=1}^{l}r_{i,k}}\cdot \prod_{k=1}^{l}\psi^{\left(r_{i,k}+d_{0}+t\right)\cdot \Lambda_{k,j}}\cdot \prod_{k=1}^{l}{\left(u_{j}\right)}^{\left(r_{i,k}+d_{0}+t\right)\cdot \Lambda_{k,j}\cdot v_{k}}\cdot \prod_{k=1}^{l}{\left(u_{j}\right)}^{d_{k}\cdot \Lambda_{k,j}\cdot v_{k}},g\right)\cdot \prod_{k=1}^{l}e\left({u_{j}}^{\eta_{k}\cdot \Lambda_{k,j}},X_{1}\right)$

$=e\left(Q_{j},g\right)\cdot \prod_{k=1}^{l}e\left({u_{j}}^{\eta_{k}\cdot \Lambda_{k,j}},X_{1}\right)$, when j=1, we have that $\sum_{k=1}^{l}\Lambda_{k,j}\cdot \eta_{k}=1$, so

$e\left(f,g_{1}\right)\cdot e\left(y,X_{3}\right)\cdot e\left(g,X_{4}\right)\cdot e\left(\vartheta^{H(M)}\cdot \psi ,X_{1}\right)\cdot e\left(\varpi ,X_{2}\right)\cdot \prod_{k=1}^{l}e\left(\psi^{\Lambda_{k,j}}\cdot {u_{j}}^{v_{k}\cdot \Lambda_{k,j}},X_{5,k}\right)\cdot \prod_{k=1}^{l}e\left({u_{j}}^{v_{k}\cdot \Lambda_{k,j}},I_{k}\right)$

$=e\left(Q_{j},g\right)\cdot e\left(u_{j},X_{1}\right)$; when j>1, we have that $\sum_{k=1}^{l}\Lambda_{k,j}\cdot \eta_{k}=0$, so$e\left(f,g_{1}\right)\cdot e\left(y,X_{3}\right)\cdot e\left(g,X_{4}\right)\cdot e\left(\vartheta^{H(M)}\cdot \psi ,X_{1}\right)\cdot e\left(\varpi ,X_{2}\right)\cdot \prod_{k=1}^{l}e\left(\psi^{\Lambda_{k,j}}\cdot {u_{j}}^{v_{k}\cdot \Lambda_{k,j}},X_{5,k}\right)\cdot \prod_{k=1}^{l}e\left({u_{j}}^{v_{k}\cdot \Lambda_{k,j}},I_{k}\right)$

$=e\left(Q_{j},g\right)$.
